# Cerebral organoids transplantation improves neurological motor function in rat brain injury

**DOI:** 10.1111/cns.13286

**Published:** 2020-02-22

**Authors:** Zhi Wang, Shu‐Na Wang, Tian‐Ying Xu, Chen Hong, Ming‐He Cheng, Peng‐Xi Zhu, Jian‐Sheng Lin, Ding‐Feng Su, Chao‐Yu Miao

**Affiliations:** ^1^ Department of Pharmacology Second Military Medical University/Naval Medical University Shanghai China; ^2^ Integrative Physiology of the Brain Arousal Systems CRNL, INSERM U1028‐CNRS UMR 5292 School of Medicine Claude Bernard University Lyon France

**Keywords:** brain injury, cerebral organoids, functional recovery, neural repair, transplantation

## Abstract

**Background and Purpose:**

Cerebral organoids (COs) have been used for studying brain development, neural disorders, and species‐specific drug pharmacology and toxicology, but the potential of COs transplantation therapy for brain injury remains to be answered.

**Methods:**

With preparation of traumatic brain injury (TBI) model of motor dysfunction, COs at 55 and 85 days (55 and 85 d‐CO) were transplanted into damaged motor cortex separately to identify better transplantation donor for brain injury. Further, the feasibility, effectiveness, and underlying mechanism of COs transplantation therapy for brain injury were explored.

**Results:**

55 d‐CO was demonstrated as better transplantation donor than 85 d‐CO, evidenced by more neurogenesis and higher cell survival rate without aggravating apoptosis and inflammation after transplantation into damaged motor cortex. Cells from transplanted COs had the potential of multilinage differentiation to mimic in‐vivo brain cortical development, support region‐specific reconstruction of damaged motor cortex, form neurotransmitter‐related neurons, and migrate into different brain regions along corpus callosum. Moreover, COs transplantation upregulated hippocampal neural connection proteins and neurotrophic factors. Notably, COs transplantation improved neurological motor function and reduced brain damage.

**Conclusions:**

This study revealed 55 d‐CO as better transplantation donor and demonstrated the feasibility and efficacy of COs transplantation in TBI, hoping to provide first‐hand preclinical evidence of COs transplantation for brain injury.

## 1 INTRODUCTION

With the evolution of stem cell technologies, increasing studies focus on the development of three‐dimensional (3D) organ‐like tissues, also known as organoids, from human‐induced pluripotent stem cells (iPSCs) or isolated organ progenitor cells in vitro.^1^ The generated organoids can mimic cytoarchitecture, cell‐cell interactions and development of in‐vivo organs, including organoids of gut, kidney, intestine, retina, and others.^1^ Thus, the successful generation of cerebral organoids (COs) is a breakthrough in the field of brain research.^2^ Unlike conventional cell culture with pure populations of particular stem cell‐derived cell types,^1^ the 3D COs generated from human PSCs contain diverse cell types, including neural progenitor cells (NPCs), neural stem cells (NSCs), mature and immature neurons, glial cells, etc They can form tissue morphology (up to 4 mm in diameter of single CO).^2^, ^3^ The COs also show robust neural connectivity and functionality to mimic in‐vivo brain development and capture features of in‐vivo brain regions.^2^, ^4^, ^5^, ^6^

Cerebral organoids and brain‐region‐specific organoids have been used for studying brain development, neural disorders, and species‐specific drug pharmacology and toxicology. For example, COs have been used for modeling human microcephaly induced by zika virus infection,^2^ autism spectrum disorders induced by CRISPR/Cas9‐mediated gene mutation,^7^ neurodegenerative microenvironment by using Alzheimer's patient‐derived hiPSCs,^8^ and developmental neurotoxicity with the exposure of rotenone,^9^ alcohol,^10^ vincristine,^11^ and tranylcypromine.^12^ There is no report of COs transplantation until the first study that establishes an in‐vivo model of vascularized human brain organoids.^13^ Transplantation of COs into retrosplenial cortex of immunodeficient mice shows progressive neural differentiation and maturation, and forms functional neuronal networks with host brain, but with no benefit on spatial learning ability.^13^ A further study demonstrates that transplanted COs have higher cell survival rate, better multilineage neurodifferentiation and robust vascularization than NSCs transplantation in the mice brain.^14^ Therefore, COs are an alternative source of NSCs as a tissue‐based transplantation donor for neural repair.

Traumatic brain injury (TBI) is a leading cause of death and disability in children and young adults worldwide. According to the World Health Organization, TBI will continue to be a major health problem and leading cause for disability by the year 2020.^15^ Although extensive researches have been done, there is still no effective therapy for TBI. In the recent decades, cell transplantation has been proved to be an effective therapy for TBI, providing a promising regenerative medical strategy.^16^, ^17^, ^18^ However, conventional cell‐based transplantation faces the hurdles of poor cell survival and inadequate neural differentiation after transplantation. Transplantation of tissue has a higher cell survival rate as compared to transplantation of cell suspension grafts,^19^ and COs transplantation has more advantages than NSCs transplantation.^14^ We wonder whether transplanted COs could repair damaged brain tissue and improve dysfunction caused by brain injury. Meanwhile, as COs at different culture stage contain diverse mixture of NPCs/NSCs and neural cells, it is still unknown which culture stage of COs is the better donor for brain injury transplantation.

Our group previously performed several studies on brain injury and NSCs.^20^, ^21^, ^22^, ^23^ Here, with preparation of rat TBI model of motor dysfunction, we transplanted COs at different culture stage separately into damaged motor cortex to identify which is the better transplantation donor and explored the feasibility, effectiveness, and underlying mechanism of COs transplantation therapy for brain injury.

## 2 MATERIALS AND METHODS

### 2.1 Animals

All Sprague‐Dawley rats (male, 250 ± 30 g) were purchased from Sino‐British SIPPR/BK Lab Animal Ltd. All rats received humane care and were kept in a 12‐hour light/dark cycle with free access to food and water throughout the study. All animal experiments were approved by the Institutional Animal Care and Use Committee of Second Military Medical University, China, and in compliance with the ARRIVE guidelines.^24^

### 2.2 Human embryonic stem cell culture

H1 human embryonic stem cells (hESCs) were obtained from WiCell and cultured with minor modifications.^25^ In the Matrigel‐coated plates (Corning, hESC‐Qualified), hESCs were cultured with mTesR™1 maintenance medium (STEMCELL, Canadian) and passaged every 4‐6 days by using TrypLE (Gibco). The use of hESCs obeys Ethical Guiding Principles for the Research of Human Embryonic Stem Cell.

### 2.3 Culture of cerebral organoids

Cerebral organoids were cultured from hESCs.^3^ hESCs were dissociated into single cells by Accutase (Gibco, MA, USA). 1.35 × 10^4^ cells per 150 µL were plated into each well of ultra‐attachment 96‐well plate (Corning) to generate embryonic bodies (EBs) with low‐bFGF hESC medium. The low‐bFGF medium contained DMEM/F12 (Invitrogen), 20% KOSR (Invitrogen), 3% hES‐quality FBS (Gibco), 1% GlutaMAX (Invitrogen), 1% MEM‐NEAA (Invitrogen), 55 μmol/L 2‐Mercaptoethanol (Merck), 4 ng/mL bFGF (Peprotech), and 50 μmol/L ROCK inhibitor Y‐27632 (Merck). After 4‐5 days, hES medium (low‐bFGF medium without bFGF and Y‐27632) was used to culture EBs. When the diameter of EBs was up to 500 μm, EBs were transferred into ultra‐attachment 24‐well plate with neural induction medium to form neuroepithelial tissue. The neural induction medium contained DMEM‐F12 with 1% N2 supplement, 1% GlutaMAX supplement, 1% MEM‐NEAA, and 1 μg/mL heparin. After 4‐5 days, neuroepithelial tissues were formed with radial organization of pseudostratified epithelium in the brighter outside of EBs‐derived tissues. Then, neuroepithelial tissues were transferred into Matrigel droplets one by one and further cultured in the 60‐mm dish with COs differentiation medium. The COs differentiation medium contained 50% DMEM/F12, 50% Neurobasal medium, 0.5% N2 supplement, 1% GlutaMAX supplement, 0.5% MEM‐NEAA, 2.8 ng/mL human insulin solution (Sigma‐Aldrich), 55 μmol/L 2‐Mercaptoethanol and 1% B27 supplement (without vitamin A). After 4‐5 days, expanded neuroepithelial tissues were transferred into spinning bioreactor in the Micro‐Stir Slow Speed Magnetic Stirrers (Wheaton) at the speed of 85 rpm for long‐term culture. The culture medium was changed with COs differentiation medium that contained 1% B27 supplement (with vitamin A).

### 2.4 Cryo‐sectioning and immunofluorescence staining of cerebral organoids

Cerebral organoids were washed with 0.1 M phosphate‐buffered saline (1× PBS) and fixed with 4% (wt/vol) PFA for 15 minutes. Then, 30% (wt/vol) sucrose solution was added to make tissue dehydration at 4°C overnight. Twelve hours later, COs were embedded in warm gelatin/sucrose solution and stored at −80°C. As for cryo‐sectioning, COs were further embedded in OCT compound (Tissue‐plus, Fisher Healthcare) and sectioned into frozen coronal slices (8 μm thickness) in the cryostat (CM3050S; Leica Microsystems). Further immunofluorescence staining of embedded COs was performed under standard procedures. Antibodies used in this study were listed in Table S1.

### 2.5 Traumatic brain injury model

Traumatic brain injury model was prepared as previous report.^26^ All rats were given Cyclosporin A (10 mg/kg, i.p.) on the day before surgery. A combination of ketamine (50 mg/kg), xylazine (2.6 mg/kg), and acepromazine (0.50 mg/kg) was used to anesthetize rats (i.p.). As for craniotomy, a longitudinal incision (approximately 4 cm) was made along brain midline. A skull window (1.5 cm length, 0.6 cm breadth) in the right skull was made without damaging brain parenchyma. Under the stereotaxic apparatus, mechanical injury was made by biopsy punch to form a cavity of 3 mm diameter and 2 mm depth in the right motor cortex of rat brain. The center of a cavity in the TBI model for biological research located at 1.5 mm lateral to the midline and 0.5 mm posterior to bregma. The centers of two connected cavities in the TBI model for studying motor functional recovery located at 1.5 mm lateral to the midline, 1 mm anterior to bregma, and 2 mm posterior to bregma respectively.

All rats were randomly grouped. The rats in the Sham group underwent craniotomy without brain injury. The rats in the TBI group were performed as aforementioned without COs transplantation. In the transplantation groups, COs at 55 and 85 days (namely 55 d‐CO and 85 d‐CO, respectively) were separately transplanted into the cavity of damaged motor cortex immediately after TBI surgery, namely 55 d‐CO transplantation and 85 d‐CO transplantation groups. The number of transplanted COs was as the same as the number of cavities that made in the motor cortex by biopsy punch. After surgery, skull window was sealed with piece of skull and bone wax. The incision was sutured and covered with erythromycin ointment to prevent infection. All operations were performed under aseptic conditions. Cyclosporine A was intraperitoneally injected every other day until rats were sacrificed. There was no animal death in the rat TBI model with or without transplantation.

### 2.6 Brain collection and immunofluorescence staining

Rat brains were harvested at the indicated day post implantation (dpi). Transcardial perfusion was performed with 4% paraformaldehyde (pH 7.4), and brains were collected carefully without disrupting lesioned site and transplanted COs and stored at –80°C. With 4% paraformaldehyde fixation at 4°C for 24 hours and tissue dehydration with 15%‐30% sucrose/paraformaldehyde, brains were embedded in OCT compound (Tissue‐plus, Fisher Healthcare) and sectioned into frozen coronal slices (8 μm thickness) in the cryostat (CM3050S; Leica Microsystems). As for immunofluorescence staining, nonspecific binding sites were blocked with 10% normal donkey serum (Jackson ImmunoResearch) for 2 hours at room temperature. Brain slices were then incubated with primary antibodies (Table S1) at 4°C overnight. After being washed by 1× PBS for three times, corresponding secondary antibodies (Alexa 488‐conjugated and Cy3‐conjugated, Table S1) were added and incubated for 2 hours at room temperature. Nuclei were stained with DAPI for 10 minutes. FLUOVIEW FV1000 Confocal laser scanning microscope (Olympus) or Pannoramic MIDI automatic digital slide scanner (3D HISTECH) was used to capture images.

BrdU immunostaining was in consistent with previous report.^27^ BrdU (Sigma‐Aldrich) was intraperitoneally injected every other day (50 mg/kg, IP, dissolved in saline). Brain slices of BrdU immunostaining were incubated with 1 N HCl at 45°C for 30 minutes and then neutralized with 0.1 mol/L sodium borate buffer (pH 8.0) before immunofluorescence staining. The positive cells of immunofluorescence staining were counted by Image J 1.5 software (Wayne Rasband, NIH) and analyzed by a blind observer. The representative images used to count the number of positive cells were taken from six random microscope fields in the transplantation periphery of ipsilateral cortex, SGZ or SVZ, and repeated with at least 3 independent animals per group.

### 2.7 Histology examination and immunohistochemistry staining

Brains were collected and used for histology examination and immunohistochemistry staining as the above.^28^ Briefly, with fixation with 4% paraformaldehyde at 4°C for 24 hours and tissue dehydration with 15%‐30% sucrose/paraformaldehyde, brains were embedded and sectioned into paraffin‐coated horizontal slices (8 μm thickness) containing brain injury sites. Then, brain slices were deparaffinized and stained with hematoxylin and eosin (HE). As for immunohistochemistry staining, brain slices were deparaffinized and retrieved antigen with citric acid buffer (PH 6.0) and blocked with 10% normal donkey serum (Jackson ImmunoResearch) for 2 hours at room temperature. Then, brain slices were incubated with specific primary antibodies (Table S1) at 4°C overnight and HRP‐conjugated secondary antibody at room temperature for 2 hours. Fresh chromogenic substrate DAB was added to visualize the section staining. Digital microscope (Leica Microsystems) was used to obtain images. The positive cells of immunohistochemistry staining were counted by Image J 1.5 software (Wayne Rasband, NIH) and analyzed by a blind observer. The representative images used to count the number of positive cells were taken from six random microscope fields in the transplantation periphery of ipsilateral cortex and repeated with at least 3 independent animals per group.

### 2.8 Tissue lysate and immunoblotting

Tissue lysates and immunoblotting were performed as standard procedure.^29^ Briefly, ipsilateral hippocampus was isolated from brain on the ice. The RIPA buffer (Beyotime Biotechnology) with protease inhibitor cocktail (Pierce) was used as lysis buffer. Protein extraction was collected after homogenate and centrifugation at 22 000 *g*, 4 ℃ for 20 minutes and stored at –80°C. The protein concentration was determined by enhanced BCA protein assay kit (Beyotime Biotechnology). After electrophoresis in 10% SDS‐PAGE, proteins were transferred into nitrocellulose membranes and incubated with primary antibodies (Table S1) and corresponding secondary antibodies conjugated with Infrared‐Dye (Li‐Cor). The images of immunoblotting were obtained in the Odyssey Infrared Fluorescence Imaging System (Li‐Cor). All immunoblotting experiments were repeated at least three times. The quantification of protein expression was analyzed in the Image J 1.5 software (Wayne Rasband, NIH) and analyzed by a blind observer.

### 2.9 Behavior tests

In the behavior test of TBI model, modified neurological severity scores (mNSS) and beam walking test were used to evaluate the recovery of neurological motor function.^30^, ^31^ All animals were blinded to experimenter in all behavior tests. The baseline of neurological motor function of these rats before operation was similar.

#### 2.9.1 mNSS evaluation

Modified neurological severity scores (mNSS) were used to evaluate rat neuromuscular function. The mNSS evaluation indices were evaluated as follows: forelimb flexion (0 score, none; 0.5 score, slightly flexion; 1.0 score, the shoulder flexion can surround the entire the forelimb flexion); twist (0 score, none; 0.5 score, slightly twist; 1.0 score, forelimbs and heads can reach the hind limbs); side push (0 score, equal on both sides; 0.5 score, the ipsilateral weakened; 1.0 score, the ipsilateral has no resistance); circle (0 score, none; 0.5 score, large circle; 1.0 score, small circle); hind limb placement (0 score, rapid recovery; 0.5 score, recovery delay; 1.0 score, no recovery); and free activity (0 score, free activity; 0.5 score, reduced activity; 1.0 score, stimulating to be active; 2.0 score, stimulation is also inactive). mNSS scores were the sum of the above indexes.

#### 2.9.2 Beam walking test

All rats were trained for one week before TBI surgery to ensure all rats can walk  through the balance beam smoothly. The balance beam used in this study was 2 cm width and 100 cm length. The beam walking test indices were evaluated as the follows: 0 score, smoothly through the balance beam without tumble; 1.0 score, smoothly cross the balance beam and less than 50% of the way with slip feet; 2.0 score, smoothly cross the balance beam and more than 50% of the way with slip feet; 3.0 sore, cross the balance beam but the ipsilateral limb does not help move forward; 4.0 score, cannot cross the balance beam but can balance on it; 5.0 score, falling from the balance beam.

### 2.10 Randomization and blinding

All animals were randomly assigned into different groups. All researchers were blind to treatment or group throughout the behavioral testing, scoring, and statistical analysis.

### 2.11 Statistical analysis

All data were shown as mean ± SEM. The line graphs were prepared in SigmaPlot 10.0 software (Systat Software Inc); the histograms were prepared in GraphPad Prism 7.0 statistical software (GraphPad Software, Inc). Statistical analyses were performed in the SPSS 11.0 software (SPSS Inc). Two‐tailed Student's *t*‐test was used for comparison between two groups. ANOVA comparison followed by Bonferroni post hoc tests was used for comparison of mean value among groups. *P* < .05 was considered statistically significant.

## 3 RESULTS

### 3.1 Comparison of cell number and composition in COs at 55 and 85 days

**Figure 1:**
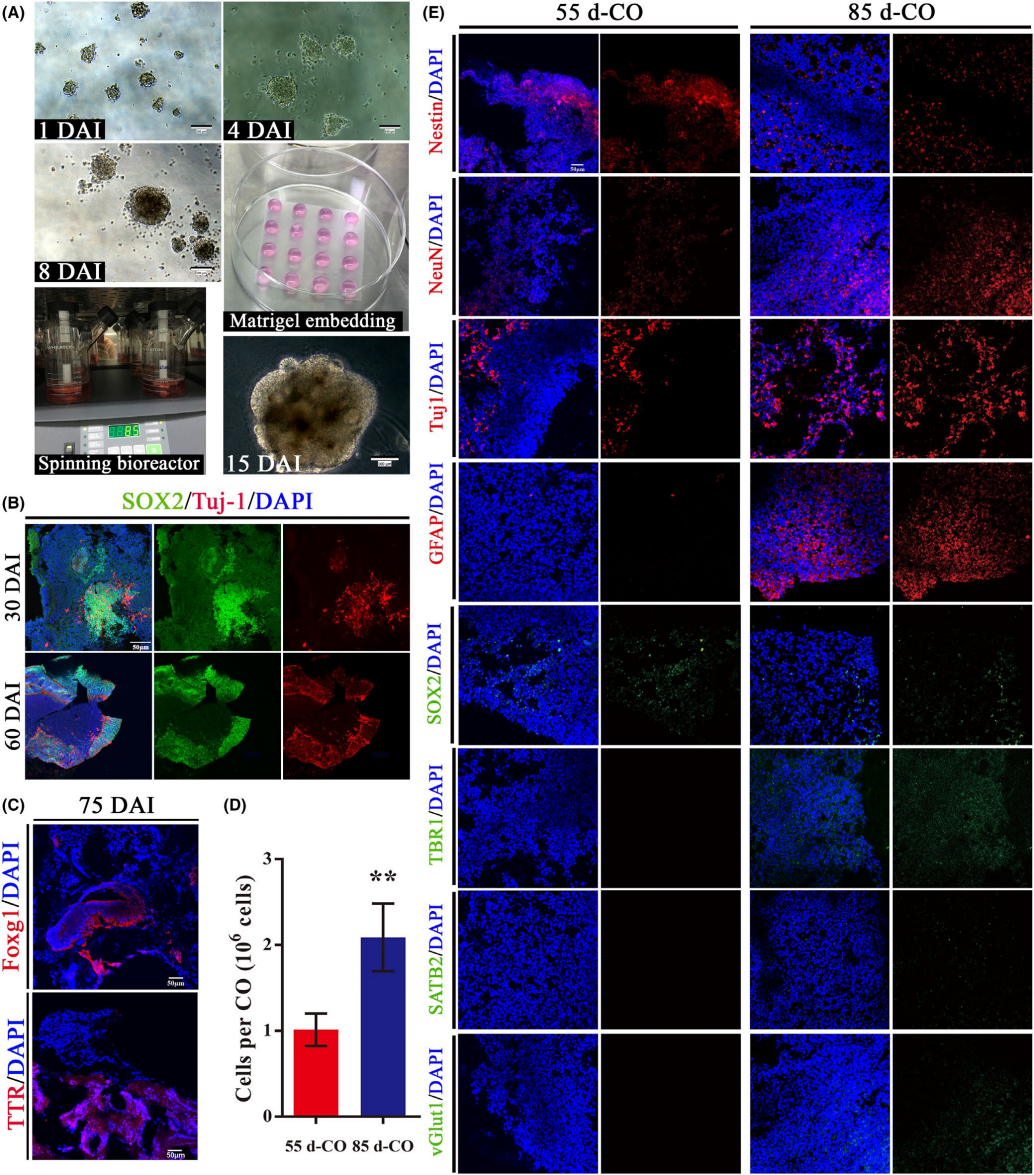
Comparison of cell number and composition in COs at 55 and 85 d. A, Schematic diagram of COs culture. The day of embryonic bodies (EBs) initially made from hESCs was defined as day 0. EBs gradually showed bright surface with relative dark center from 1 to 8 d after induction (DAI). After Matrigel embedding for expanding neuroepithelial buds, well‐defined polarized neuroepithelium‐like structures resembled neural tubes at 15 DAI. Scale bars: 100 μm. B, Immunostaining for neural progenitor cells (SOX2, green) and neurons (Tuj1, red) in COs at 30 and 60 DAI. DAPI labels nuclei (blue). Scale bar: 100 μm. C, Immunostaining for COs at 75 DAI with forebrain (Foxg1, red) and choroid plexus (TTR, red). Scale bars: 50 μm. D, Cell number in COs at 55 and 85 d after induction (namely 55 d‐CO and 85 d‐CO). ^**^ *P* < .01. All data were shown as mean ± SEM and analyzed by Student's *t*‐test, n = 8. E, Cell composition in 55 d‐CO and 85 d‐CO. Nestin (red), neural stem cells; NeuN (red), mature neurons; Tuj1 (red), neurons; GFAP (red), astrocytes; SOX2 (green), neural progenitor cells; TBR1 (green), preplate/deep‐layer neurons; SATB2 (green), surface‐layer neurons; vGlut1 (green), excitatory glutamatergic neurons. Scale bar: 50 μm

Embryonic bodies (EBs) underwent germ layer differentiation and neural induction as shown at 1 and 4 days after induction (DAI) (Figure 1A). EBs at 8 DAI showed ectodermal differentiation with evidence of brightened surface and relative dark center. After Matrigel embedding for expanding neuroepithelial buds, neuroepithelium‐like structures were formed to resemble neural tubes at 15 DAI (Figure 1A). Subsequently, neuroepithelium‐like structures in Matrigel droplets were transferred to spinning bioreactor for further growth of cerebral tissues. As expected, the expression of neurons (Tuj‐1) was gradually upregulated and the expression of NPCs (SOX2) was gradually downregulated during the culture of COs (Figure 1B). COs at 75 DAI showed positive expression of forebrain (Foxg1) and choroid plexus (TTR), indicating the successful formation of cerebral cortical morphology and brain regional identities (Figure 1C).

Considering different culture stages of COs contain diverse neural cell types, we characterized and compared cell number and composition of COs at 55 and 85 DAI (namely 55 d‐CO and 85 d‐CO) that were used for transplantation in our study (Figure 1D,E). The cell number in 85 d‐CO was twice in 55 d‐CO (2.09 ± 0.13 vs 1.01 ± 0.07 × 10^6^ cells per CO, n = 8) (Figure 1D). 55 d‐CO showed higher expression of NPCs (SOX2) and NSCs (Nestin), but lower expression of neurons (NeuN, Tuj‐1), astrocytes (GFAP), preplate/deep‐layer neurons (TBR1), late‐born superficial neurons (SATB2), and glutamatergic neurons (vGlut1) than 85 d‐CO (Figure 1E). These results indicate 85 d‐CO is more mature than 55 d‐CO, in accordance with development process of in‐vitro cultivation of COs. Next, 55 d‐CO and 85 d‐CO were used as transplantation donors to compare the effects of transplantation on neurogenesis and cell survival in a rat brain injury model.

### 3.2 55 d‐CO is a better transplantation donor than 85 d‐CO for neurogenesis and cell survival in rat brain injury

**Figure 2:**
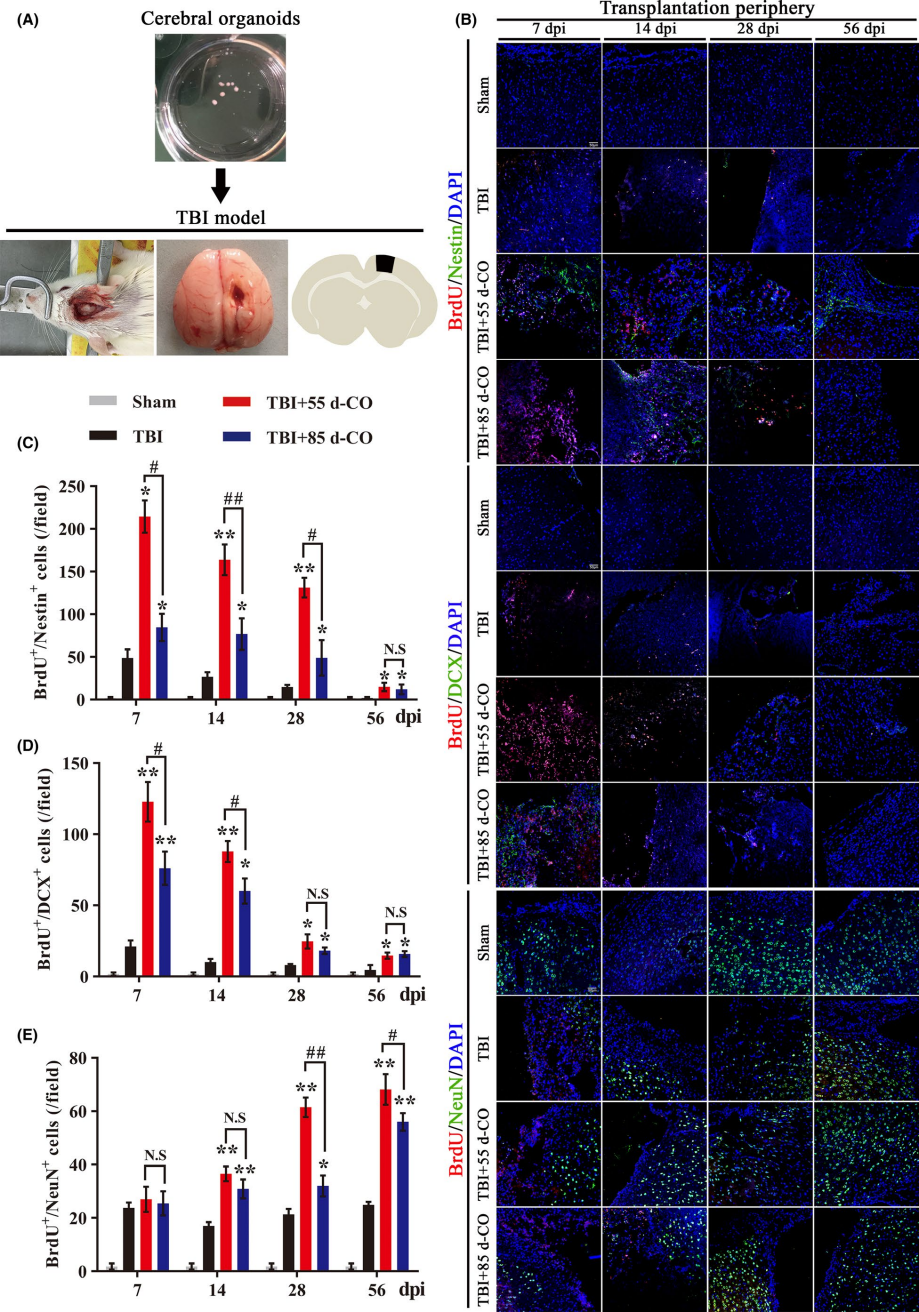
More neurogenesis in 55 d‐CO than 85 d‐CO transplantation periphery of ipsilateral cortex in rat TBI model. A, Illustration of COs transplantation into damaged motor cortex in the rat TBI model. The lesioned cavity was made at the motor cortex (1.5 mm lateral to midline, 0.5 mm posterior to bregma) by biopsy punch. B, Representative images of neurogenesis in the transplantation periphery of ipsilateral cortex by immunostaining of proliferated neural stem cells (BrdU^+^/Nestin^+^, red and green, respectively), migrated newborn neurons (BrdU^+^/DCX^+^, red and green, respectively), and differentiated mature neurons (BrdU^+^/NeuN^+^, red and green, respectively) at 7, 14, 28, and 56 dpi in Sham, TBI, 55 d‐CO transplantation, and 85 d‐CO transplantation groups. BrdU, proliferation marker. DAPI labels nuclei (blue). Scale bars: 50 μm. C‐E, Quantitative analysis of neurogenesis by counting BrdU^+^/Nestin^+^, BrdU^+^/DCX^+^, and BrdU^+^/NeuN^+^ cells in the transplantation periphery of ipsilateral cortex at 7, 14, 28, and 56 dpi. Immuno‐stained positive cells were counted with six random microscope fields in the transplantation periphery of ipsilateral cortex, and repeated with at least three independent animals per group. All data were shown as mean ± SEM and analyzed by ANOVA with Bonferroni posthoc tests. *P < .05, **P < .01 vs TBI group; #*P* < .05, ##*P* < .01. N.S, not significant

At 7, 14, 28, and 56 day post implantation (dpi) of COs into damaged motor cortex (Figure 2A), immunostaining was used to detect brain neurogenesis, namely BrdU^+^/Nestin^+^ proliferated NSCs, BrdU^+^/DCX^+^ migrated newborn neurons, and BrdU^+^/NeuN^+^ differentiated mature neurons. BrdU^+^/Nestin^+^, BrdU^+^/DCX^+^, and BrdU^+^/NeuN^+^ cells in transplantation periphery of ipsilateral cortex in 55 d‐CO and 85 d‐CO transplantation groups were significantly more than those in Sham and TBI groups, suggesting the proproliferation and prodifferentiation effects of COs transplantation (Figure 2B‐E). Subgranular zone (SGZ) of hippocampus and subventricular zone (SVZ) of lateral ventricles are known areas of neurogenesis.^32^, ^33^ Detection of neurogenesis in these two areas showed similar effects. BrdU^+^/Nestin^+^ and BrdU^+^/DCX^+^ cells were more in ipsilateral SGZ and SVZ in 55 d‐CO and 85 d‐CO transplantation groups than those in Sham and TBI groups, indicating enhanced neurogenesis by COs transplantation (Figures S1‐S2).

To determine which culture stage of COs has better proproliferation and prodifferentiation effects, we compared neurogenesis between 55 d‐CO and 85 d‐CO transplantation groups. Compared to 85 d‐CO transplantation group, 55 d‐CO transplantation group had more BrdU^+^/Nestin^+^ and BrdU^+^/DCX^+^ cells at 7, 14 dpi and more BrdU^+^/NeuN^+^ cells at 28, 56 dpi in transplantation periphery of ipsilateral cortex (Figure 2B‐E). BrdU^+^/Nestin^+^ and BrdU^+^/DCX^+^ cells in ipsilateral SGZ and SVZ of 55 d‐CO transplantation group were also more than those in 85 d‐CO transplantation group (Figures S1‐S2). Although COs transplantation enhanced neurogenesis in contralateral SGZ and SVZ, neurogenesis between 55 d‐CO and 85 d‐CO transplantation groups had no obvious difference (Data not shown). These effects indicate that both 55 d‐CO and 85 d‐CO transplantation donors enhance neurogenesis with proproliferation and prodifferentiation effects in the rat TBI model, and 55 d‐CO has better effect in promoting neurogenesis than 85 d‐CO after transplantation into damaged motor cortex.

**Figure 3:**
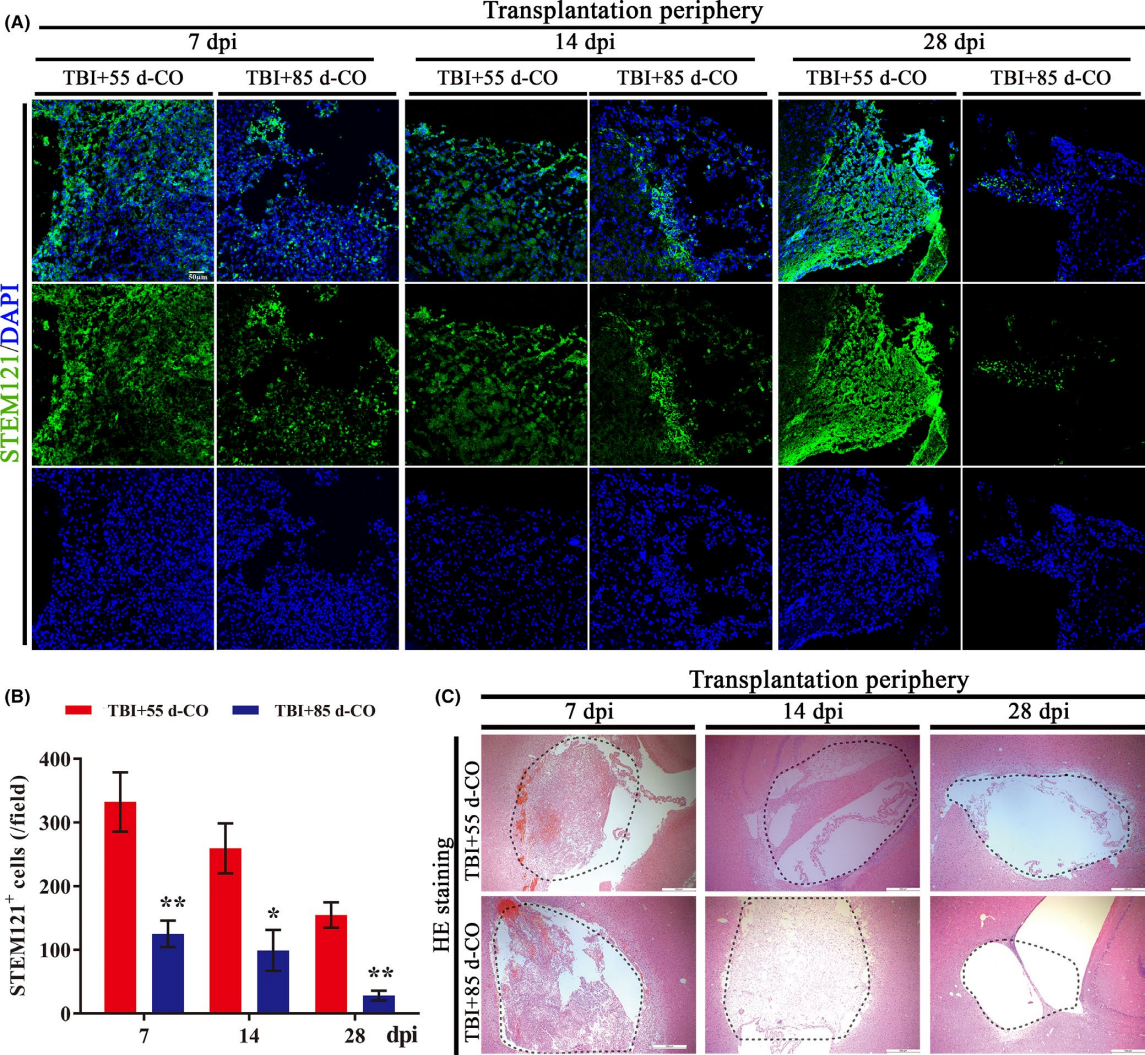
More cell survival from transplanted 55 d‐CO than 85 d‐CO in rat TBI model. A, Representative images of COs survival by immunostaining of human cytoplasmic marker (STEM121, green) at 7, 14 and 28 dpi in the transplantation periphery of ipsilateral cortex of 55 d‐CO transplantation and 85 d‐CO transplantation groups. STEM121^+^ cells distributed throughout the lesioned cavity. DAPI labels nuclei (blue). Scale bar: 50 μm. B, Quantitative analysis of STEM121^+^ cells per field in 55 d‐CO transplantation and 85 d‐CO transplantation groups. Immunostained positive cells were counted with six random microscope fields in the transplantation periphery of ipsilateral cortex and repeated with at least three independent animals per group. All data were shown as mean ± SEM and analyzed by ANOVA with Bonferroni posthoc tests. *P < .05 and **P < .01 vs 55 d‐CO transplantation group. C, Representative images of COs survival by HE staining of horizontal sections with cavity in rat ipsilateral cortex. The cavity was filled with transplanted COs (dotted black lines). Scale bars: 500 μm

We further compared cell survival between 55 d‐CO and 85 d‐CO transplantation groups by immunostaining STEM121, which can be used as human cytoplastic marker of transplanted COs (Figure 3A). Although cell number was more in 85 d‐CO than 55 d‐CO and the morphological volume was larger in 85 d‐CO than 55 d‐CO before transplantation (Figure 1D), 55 d‐CO transplantation had more cell survival number than 85 d‐CO transplantation (Figure 3B). The more cell survival number in 55 d‐CO transplantation group was also confirmed by HE staining (Figure 3C), suggesting higher cell survival rate of 55 d‐CO transplantation in brain injury.

Meanwhile, we detected neural apoptosis and neuroinflammation in transplantation periphery of ipsilateral cortex among Sham, TBI, 55 d‐CO, and 85 d‐CO transplantation groups. With immunostaining of apoptotic cells (cleaved‐caspase‐3) and pro‐inflammatory cytokines [intercellular cell adhesion molecule‐1 (ICAM‐1), tumor necrosis factor α (TNF‐α), and interleukin‐1β (IL‐1β)], there was no difference in the number of apoptotic cells and pro‐inflammatory cytokines among TBI, 55 d‐CO, and 85 d‐CO transplantation groups (Figures S3‐S4). There was also no difference in rat serum ICAM‐1 concentration among Sham, TBI, and 55 d‐CO transplantation groups (Figure S4C). Therefore, both 55 d‐CO and 85 d‐CO transplantation did not aggravate neural apoptosis and neuroinflammation after brain injury.

Taken together, 55 d‐CO is a better transplantation donor for brain injury, which has more neurogenesis and more cell survival number than 85 d‐CO after transplantation into damaged motor cortex. 55 d‐CO was used for the following in‐depth transplantation study.

### 3.3 Vascularization between transplanted COs and host brain in rat brain injury

Previous study demonstrated the growth of vascular network between grafted COs and retrosplenial cortex of host brain.^13^ As expected in the present study, there was vessel formation between transplanted COs and host brain by immunostaining STEM121 with endothelial markers CD31 or CD105 (also known as endoglin) (Figure S5A). The CD31^+^ endothelial cells showed overlap with STEM121^+^ cells in transplantation periphery of ipsilateral cortex, proving the vascularization between transplanted COs and host brain (Figure S5A). Vessel formation in transplanted COs was also confirmed by immunohistochemistry staining CD31 (Figure S5B). The vascularization between transplanted COs and host brain plays a vital role in cells survival and further differentiation of transplanted COs.

### 3.4 Cells from transplanted COs have the potential of multilineage differentiation to mimic brain cortical development and support motor cortex region‐specific reconstruction via in situ differentiation and cell replacement in rat brain injury

**Figure 4:**
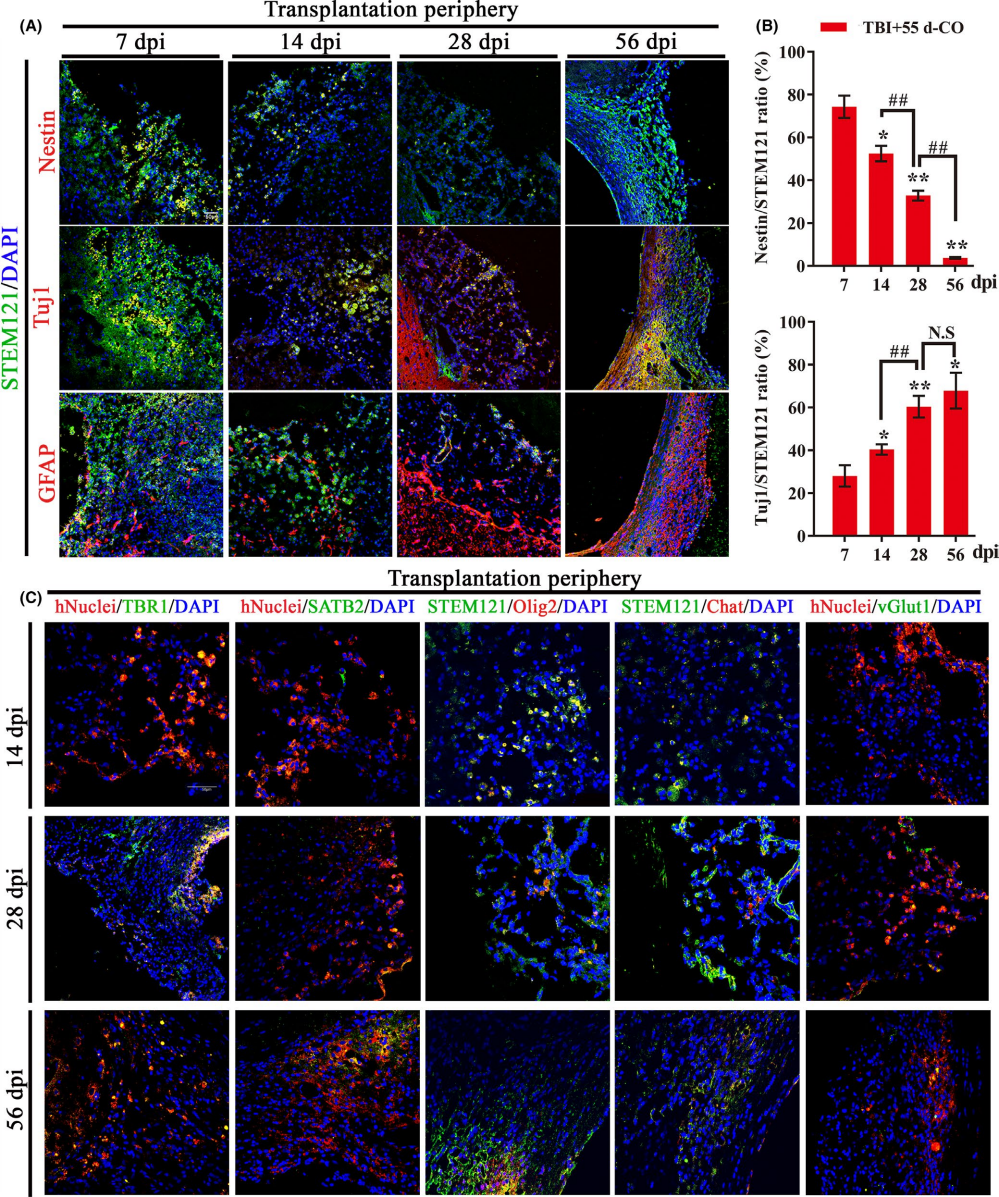
Cells from transplanted COs have the potential of multilineage differentiation to mimic brain cortical development and support motor cortex region‐specific reconstruction in rat TBI model. A, Representative images of in‐vivo differentiated COs by immunostaining of human cytoplasmic marker (STEM121, green) and neural stem cells (Nestin, red), neurons (Tuj1, red), or astrocytes (GFAP, red) at 7, 14, 28, and 56 dpi in 55 d‐CO transplantation group. The cells of STEM121^+^/Tuj1^+^ and STEM121^+^/GFAP^+^ gradually increased but STEM121^+^/Nestin^+^ cells gradually decreased until they disappeared. DAPI labels nuclei (blue). Scale bar: 50 μm. B, Quantification of the percentage of STEM121^+^/Nestin^+^ and STEM121^+^/Tuj1^+^ cells in the in‐vivo differentiated COs. Immunostained positive cells were counted with six random microscope fields in the transplantation periphery of ipsilateral cortex and repeated with at least three independent animals per group. All data were shown as mean ± SEM and analyzed by ANOVA with Bonferroni posthoc tests. *P < .05 and **P < .01 vs 55 d‐CO transplantation group at 7 dpi; ^##^ *P* < .01. N.S, not significant. C, Representative images of transplanted COs at 14, 28, and 56 dpi. Immunostaining for human cells by STEM121 (green) with motor neuronal progenitor cells (Olig2, red) and cholinergic neurons (Chat, red), and by hNuclei (red) with preplate/deep‐layer neurons (TBR1,green), surface‐layer neurons (SATB2, green), and glutamatergic neurons (vGlut1, green) showed in situ differentiation and cell replacement of transplanted COs in the damaged motor cortex. Scale bar: 50 μm

Stem cell‐based transplantation has been reported to support brain‐region‐specific reconstruction, functional replacement, and neural connection.^34^, ^35^ With immunostaining of human cell‐derived NSCs (STEM121^+^/Nestin^+^), neurons (STEM121^+^/Tuj1^+^), and astrocytes (STEM121^+^/GFAP^+^) (Figure 4A), we detected the differentiation trend of transplanted COs. The expression of neurons and astrocytes gradually increased while NSCs gradually decreased until disappeared in transplanted COs (Figure 4A,B). Compared to cell composition of 55 d‐CO before transplantation (Figure 1E), the gradually increased neurons, astrocytes, and decreased NSCs in transplanted COs of motor cortex resembled in‐vivo differentiation and maturation of brain cortical development (Figure 4A,B).

As the brain injury site was made in motor cortex, we further explored whether transplanted COs differentiated into cortical‐specific neurons and even motor neural cell linages. Cortical differentiation of transplanted COs was confirmed by TBR1 (preplate/deep‐layer neurons marker) and SATB2 (late‐born surface‐layer neurons marker) immunostaining (Figure 4C). TBR1 is involved in cortical lamination and affects the processes of cell migration and neural differentiation,^36^ and SATB2 is necessary for establishment of cortical neuronal connections.^37^ The positive expression of TBR1 and SATB2 suggests the formation of cortical layer neurons in transplanted COs. The presence of Olig2 in the early and later stages of COs differentiation reveals the cell fates of motor progenitors in the brain injury site of host brain. Immunostaining for motor progenitor cells (Olig2) proved positive expression of motor cell linage in transplanted COs (Figure 4C). Due to extensive neuronal expression in transplanted COs (Figure 4A), we wondered whether there were neurons involving the regulation of neurotransmitter release. Immunostaining for Chat (cholinergic neurons) and vGlut1 (glutamatergic neurons) confirmed the formation of cholinergic and glutamatergic neurons in transplanted COs, suggesting the formation of neurotransmitter‐related neurons (Figure 4C). These results demonstrate COs transplantation has the potential of multilineage differentiation to mimic in‐vivo brain cortical development, support motor cortex region‐specific reconstruction, and form neurotransmitter‐related neurons via in situ differentiation and cell replacement in the damaged motor cortex of rat TBI model.

### 3.5 Cells from transplanted COs show extensive migration into cortex, thalamus, and hippocampus along corpus callosum in rat brain injury

**Figure 5:**
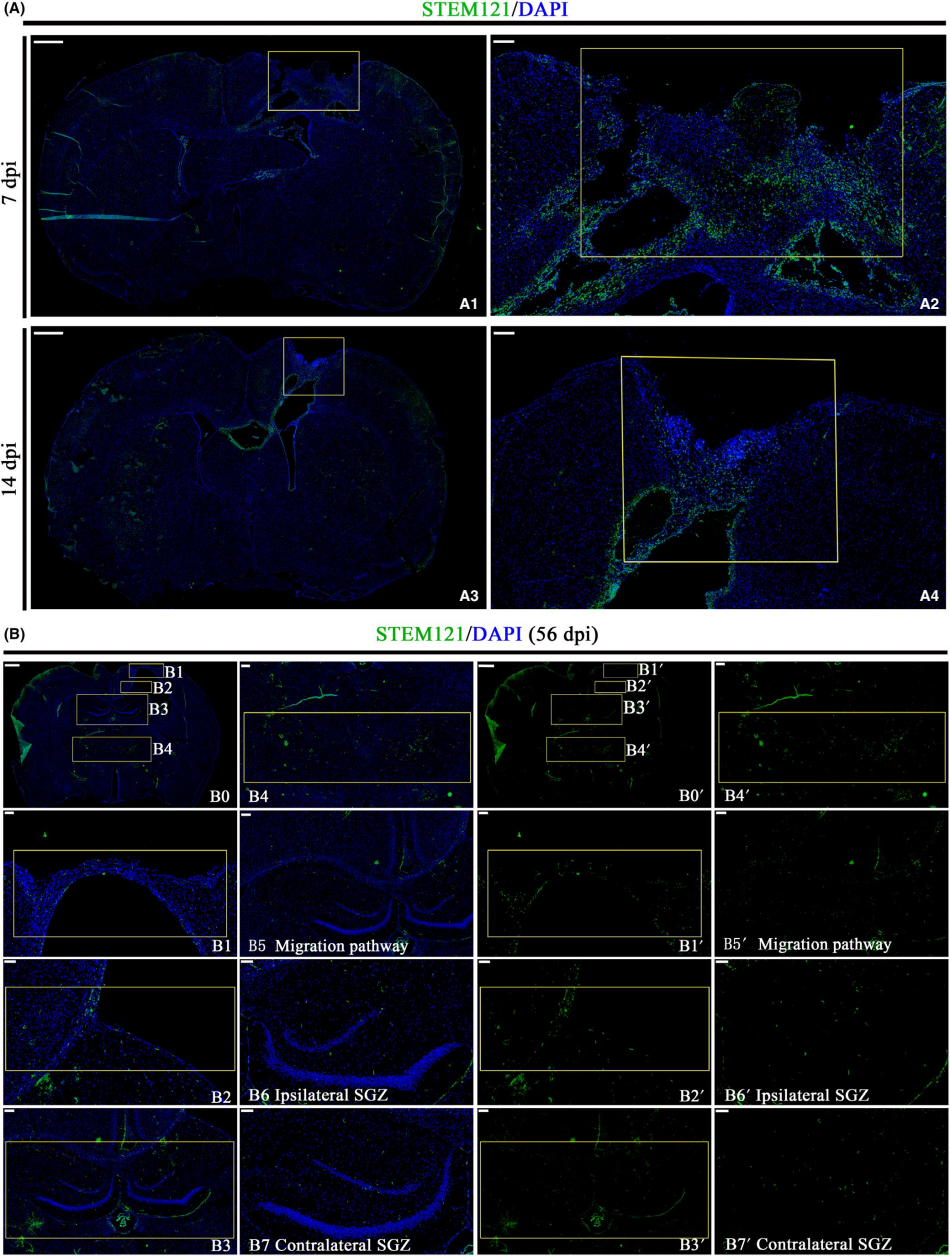
Cells from transplanted COs migrate into cortex, thalamus, and hippocampus along corpus callosum in rat TBI model. A, Representative images of whole brain scan with COs transplantation at 7 and 14 dpi in 55 d‐CO transplantation group. STEM121 (green), human cytoplasmic marker. Tissues inside the rectangle frame indicate transplanted COs in the host brain, wherein the right image is the high‐magnification view of boxed area in the left image. DAPI labels nuclei (blue). Scale bars: 1000 μm in A1;A3; 200 μm in A2;A4. B, Representative images of migration of cell from transplanted COs into host brain at 56 dpi in 55 d‐CO transplantation group. (B0 and B0′) Overall view of migration of cell from transplanted COs in rat brain. (B2 and B2′, B5 and B5′) Images showed corpus callosum as migration pathway of cells from transplanted COs into host brain. Cells from transplanted COs showed migration into cortical region (B1 and B1′), and migration into ipsilateral and contralateral hippocampus (B3 and B3′), thalamic nucleus (B4 and B4′), ipsilateral, and contralateral SGZ (B6 and B6′, B7 and B7′) in the host brain. Scale bars: 1000 μm in B0,B0′; 200 μm in B3,B3′,B4,B4′,B5,B5′; 100 μm in B1,B1′,B2,B2′,B6,B6′,B7,B7′

As there was decreased cell survival number of transplanted COs in the host brain over time (Figure 3B), we wondered whether the decreased trend was caused by cell migration of transplanted COs into host brain. Immunostaining for human cytoplastic marker STEM121 demonstrated cells from transplanted COs distributed throughout the lesioned cavity at 7, 14, 28, and 56 dpi in ipsilateral cortex of host brain (Figure 5). Cells from transplanted COs migrated into extensive regions of host brain along corpus callosum at 56 dpi and showed positive expression in ipsilateral cortical region (Figure 5B1‐B3,B1′‐B3′), ipsilateral and contralateral thalamus (Figure 5B4, 5B4′), and hippocampus (Figure 5B3, B5‐B7, 5B3′, 5B5′‐B7′). Immunostaining for hNuclei, a human nuclear marker (another human cell marker different from human cytoplastic marker STEM121), proved the same cell migration of transplanted COs in the host brain (Figure S6). Moreover, cells from transplanted COs existed in ipsilateral and contralateral SVZ of host brain (Figure S7). Therefore, cells from transplanted COs have ability to migrate into extensive regions of host brain, suggesting potential integration and connection between transplanted COs and host brain.

### 3.6 COs transplantation upregulates hippocampal neural connection proteins and neurotrophic factors in rat brain injury

**Figure 6:**
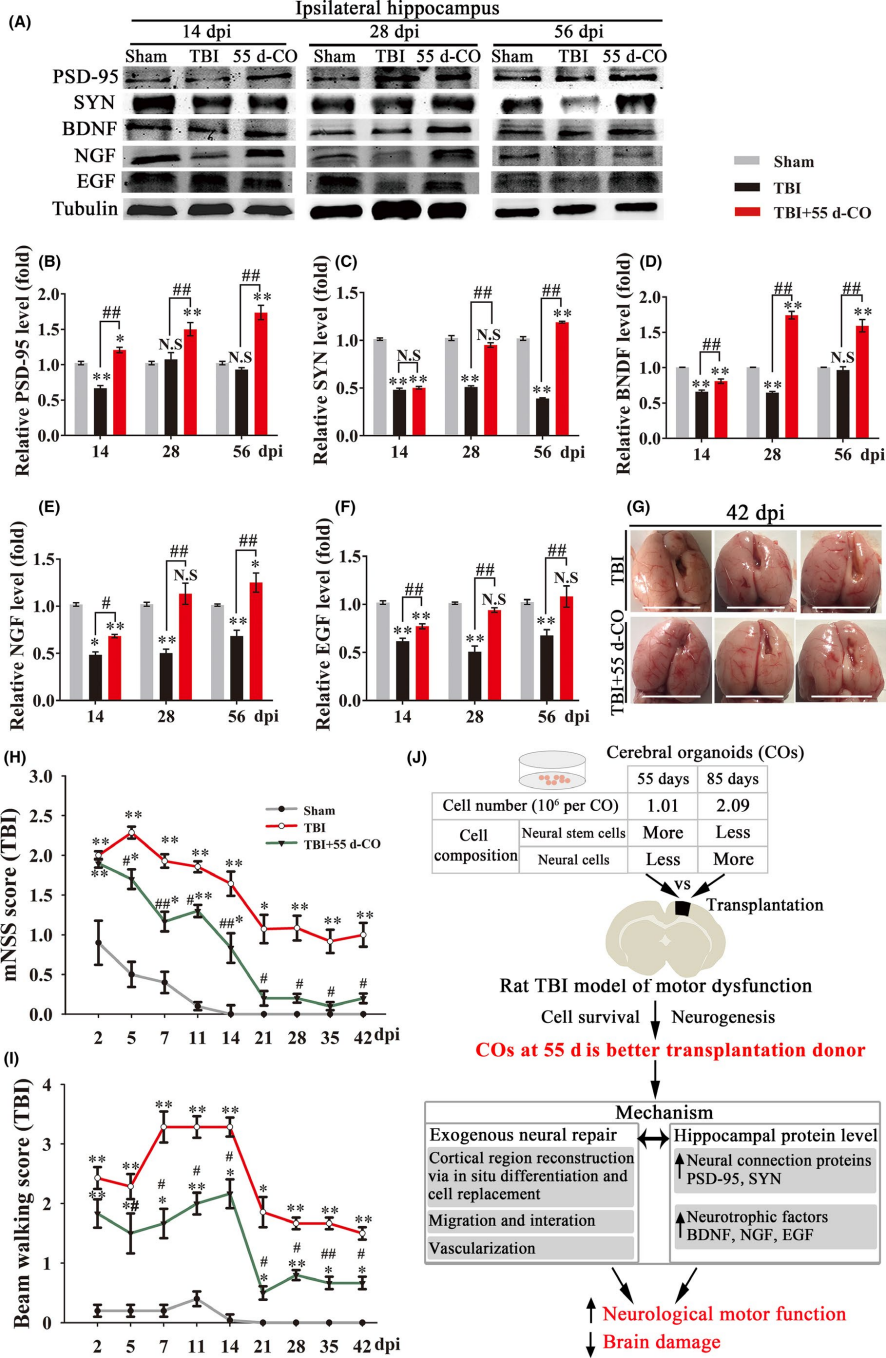
Cerebral organoids (COs) transplantation upregulates hippocampal neural connection proteins and neurotrophic factors, improves neurological motor function, and reduces brain damage in rat TBI model. A, Representative immunoblots of postsynaptic density protein 95 (PSD95, postsynaptic marker), synaptophysin (SYN, presynaptic marker), brain‐derived neurotrophic factor (BDNF), nerve growth factor (NGF), epidermal growth factor (EGF), and Tubulin (internal control protein as loading control) proteins at 14, 28, and 56 dpi in rat ipsilateral hippocampus of COs transplantation group. B‐F, Quantitative analysis of protein expressions of PSD95, SYN, BDNF, NGF, and EGF normalized to Tubulin at 14, 28, and 56 dpi in rat ipsilateral hippocampus of COs transplantation group. All immunoblotting experiments in each group were repeated with four times. All data were mean value that normalized to protein expression in Sham group. G, Representative images of whole brain at 42 dpi in the rat TBI and TBI transplanted with COs groups. The cavity in transplantation group was smaller than that in TBI group. Scale bars: 1 cm. H‐I, Rat mNSS score and beam walking test performance were recorded at 2, 5, 7, 11, 14, 21, 28, 35, and 42 dpi in rat TBI model. All data were shown as mean ± SEM and analyzed by ANOVA with Bonferroni posthoc tests, n = 8. *P < .05 and **P < .01 vs Sham group; ^#^ *P* < .05 and ^##^ *P* < .01 vs TBI group. N.S, not significant. J, The proposed mechanism for the efficacy of COs transplantation in brain injury therapy. Cell number and composition are different in 55 d‐CO and 85 d‐CO. 55 d‐CO is a better transplantation donor than 85 d‐CO for cell survival and neurogenesis. Intracerebral transplantation of 55 d‐CO into damaged motor cortex can improve neurological motor function and rescue brain damage via activation of exogenous neural repair and upregulation of neural connection proteins and neurotrophic factors

We further explored possible mechanism by detecting hippocampal protein expression of neural connection proteins and neurotrophic factors, which are tightly associated with neurogenesis and neural function. COs transplantation significantly upregulated neural connection proteins and neurotrophic factors as compared to TBI group (Figure 6A‐F). Compared to Sham and TBI groups, COs transplantation significantly upregulated postsynaptic density protein 95 (PSD‐95, postsynaptic marker) and synaptophysin (SYN, presynaptic marker), suggesting enhanced neural connection by COs transplantation after brain injury (Figure 6A‐C). The expression of neurotrophic factors brain‐derived neurotrophic factor (BDNF), nerve growth factor (NGF), and epidermal growth factor (EGF) at 14 dpi in transplantation group was higher than that in TBI group (Figure 6A,D‐F). Compared to Sham group, COs transplantation upregulated BDNF and NGF from 28 and 56 dpi, respectively, and recovered EGF to normal level at 28 dpi (Figure 6A,D‐F), proving more neurotrophic factors in the hippocampal of COs transplantation group. The upregulated neural connection proteins and neurotrophic factors mediated by COs transplantation may contribute to the enhanced neurogenesis and exogenous neural repair after brain injury.

### 3.7 COs transplantation improves neurological motor function and reduces brain damage in rat brain injury

We next explored whether transplantation of 55 d‐CO into damaged motor cortex could improve motor functional recovery and rescue brain damage. mNSS method was introduced to evaluate rat neurological function after TBI. Improved mNSS score was found from 5 dpi in transplantation group as compared to TBI group (Figure 6H). Notably, COs transplantation recovered neurological function to normal level from 21 dpi, wherein no difference was found between Sham and transplantation groups (Figure 6H). Beam walking test confirmed the improvement of motor function in COs transplantation group as compared to TBI group (Figure 6I). At 42 dpi, we observed obvious brain atrophy in the damaged ipsilateral hemisphere of TBI group (Figure 6G). Brain parenchyma in ipsilateral hemisphere of COs transplantation group showed relatively smooth and plump morphology with smaller cavity, proving the amelioration of brain damage (Figure 6G). These results provide positive preclinical evidence for functional and morphological improvement of COs transplantation in brain injury therapy.

## 4 DISCUSSION

The present study demonstrates the feasibility, effectiveness, and mechanism of COs transplantation for brain injury therapy (Figure 6J). COs transplantation can enhance neurogenesis without aggravating cell apoptosis and neuroinflammation, and 55 d‐CO is demonstrated as a better transplantation donor than 85 d‐CO, evidenced by more neurogenesis and more cell survival after transplantation into damaged cortex of rat TBI model. Cells from transplanted COs have the potential of multilinage differentiation to mimic in‐vivo brain cortical development, support region‐specific reconstruction of damaged motor cortex, form neurotransmitter release‐related neurons, and meanwhile migrate into different brain regions. Moreover, COs transplantation upregulates neural connection proteins and neurotrophic factors in ipsilateral hippocampus and improves neurological motor function and reduces brain damage after TBI.

### 4.1 The selection of research model for COs transplantation

Transplanted NSCs can reduce brain damage, enhance neural repair, provide trophic support, and improve functional recovery after brain injury.^16^, ^17^, ^31^ As COs transplantation has higher cell survival rate, better multilineage neurodifferentiation, and robust vascularization than NSCs transplantation in the mouse brain,^14^ we wondered the effect of COs transplantation on the repair of brain injury. So far, only two studies report COs transplantation and demonstrate the survival, differentiation, and vascularization of transplanted COs in retrosplenial cortex or frontoparietal cortex of mouse brain.^13^, ^14^ Considering the high disability and mortality of TBI and high incidence of TBI‐induced motor dysfunction worldwide, here we made a direct mechanical cavity in the rat motor cortex to prepare TBI model of motor dysfunction and further explore the feasibility and potential mechanism of COs transplantation. In our study, the rat TBI model of motor dysfunction has advantages that: the cavity used for accommodating transplanted COs has abundant blood supply, contributing to the survival of transplanted COs; the model has no obvious change of neuroinflammation, providing a pure and simple model to mainly study the change of transplanted COs in the host brain; the simple model has performance of motor dysfunction, supporting the study of COs transplantation on neurological motor function recovery; the rats used for TBI model have no immunodeficiency to approach clinical practice.

### 4.2 The identification of better COs transplantation donor for brain injury

Transplantation donor is an important parameter for preclinical transplantation study.^17^ Current cell transplantation studies mainly use single type of NSCs or neural cells to repair brain injury.^17^ However, the damaged tissues after brain injury contain diverse cell types rather than single cell types. COs consist of abundant neural cell types, representing an alternative transplantation donor for brain injury. As COs at different developmental stages are diverse in neural cell types, regional identities, and cell number (Figures 1A‐E and 6J), choosing 55 d‐CO or 85 d‐CO for intracerebral transplantation is a question to be answered. With comparison of neurogenesis and cell survival number after transplantation into damaged cortex, 55 d‐CO is demonstrated as a better transplantation donor for brain injury. Notably, the effectiveness of cell transplantation is related to cell transplantation dose, and it might be invalid if the cell transplantation dose is lower than 10^6^.^38^, ^39^ Thus, we did not attempt to transplant COs with shorter culture time in this study, as we found the cell number of 55 d‐CO is about 1.01 × 10^6^. Besides, both 55 d‐CO and 85 d‐CO transplantation do not aggravate neural apoptosis and neuroinflammation in transplantation periphery of ipsilateral cortex after brain injury, demonstrating the compatibility between transplanted COs and host brain. The unchanged neuroinflammation by COs transplantation may be due to the use of immunosuppressive agent cyclosporin A throughout the study. Cyclosporin A has been widely used in clinical transplantation and reported to inhibit neuroinflammation after brain injury.^40^

### 4.3 The potential mechanism of COs transplantation for brain injury

In addition to above mentioned neurogenesis, cell apoptosis, and neuroinflammation mediated by COs transplantation, we further explored the potential mechanism of transplanted COs in brain injury. As expected, there is vascular connection between transplanted COs and host brain, which is important for promoting the survival of transplanted COs. According to recently reported article, the source of blood vessels was from the host brain.^13^ The in‐vivo survival and differentiation of transplanted stem cells are important elements for functional repairment. As the transplantation site is in the motor cortex, we successively detected the trend of in‐vivo differentiation and identification of cortical layer neurons and motor neural cell lineage in transplanted COs. Transplanted COs mimic in‐vivo differentiation of brain cortical development and show positive expression of cortical layer neurons (preplate/deep‐layer and surface‐layer neurons), motor progenitor cells, and further form glutamatergic and cholinergic neurons that are related to neurotransmitter release. It is worth mentioning that the differentiation trend of transplanted COs within host brain may need to be further explored. For example, whether the differentiation proportion of neuron/gliocyte and cholinergic neuron/noncholinergic neuron is close to brain physiology needs to be further clarified. Besides, cells from transplanted COs show extensive migration into cortex, thalamus, and hippocampus along corpus callosum after brain injury. The neuronal activity and functional connectivity between transplanted COs and host brain have been proved by electrophysiological recording and optogenetics.^13^ Thus, the vascularization, differentiation, and migration of transplanted COs in the host brain support motor cortex region‐specific reconstruction after brain injury.

Both central nervous system and peripheral circulatory system have a series of physiological reactions, including excitotoxicity, oxidative stress, mitochondrial dysfunction, and so on, during chronic TBI.^41^ All these processes exacerbate the survival environment of endogenous NSCs and transplanted cells, which limit neurogenesis of endogenous NSCs.^42^ Several studies have demonstrated transplanted cells in the host brain can improve brain microenvironment, promote neurogenesis, and restore neural connectivity through paracrine role, which in turn contribute to suitable living environment for the survival of endogenous and exogenous NSCs.^43^, ^44^ In our study, we found COs transplantation upregulates expression of neural connection proteins and neurotrophic factors in ipsilateral hippocampus. The upregulation of neural connection proteins and neurotrophic factors in transplantation group may partly result from paracrine functions of transplanted COs. Thus, COs transplantation mediates dual function in promoting endogenous and exogenous neural repair. Remarkably, COs transplantation significantly recovers neurological function to normal level, improves motor function, and reduces brain damage after brain injury, providing direct evidence for functional and morphological improvement of brain injury.

In the future study, many questions remain to be answered to explore the specific action mechanisms, optimal dose and delivery routes, therapeutic time window, and safety concerns of transplanted COs for brain injury therapy. Transplantation of modified cells with overexpression of growth or trophic factors is one way to improve the survival of grafts.^45^, ^46^ By introducing genetic manipulation or providing a scaffold environment for transplanted cells,^47^, ^48^ enhancing responsiveness and sensitivity of transplanted cells to endogenous signaling is another way to increase effectiveness. It is noteworthy that the culture of COs is based on the self‐organization of hiPSCs, leading to the heterogeneity of each COs in cell composition, cell number, and so on.^3^ The heterogeneity of COs may increase uncontrollability for transplantation therapy. As the recently cultured brain‐region‐specific COs has lower heterogeneity,^4^ whether it is the better transplantation donor for brain‐region‐specific injury needs to be explored in the further study. In addition, the proliferation of NPCs/NSCs within transplanted COs needs to be controlled because of the possibility of tumorigenesis after transplantation, though we did not observe the phenomenon in our study.

## 5 CONCLUSIONS

With identification of better transplantation donor of COs, the study gives the first demonstration of feasibility, effectiveness, and mechanism of COs transplantation in motor cortex brain injury, hoping to provide first‐hand preclinical evidence of COs transplantation therapy for brain injury.

## CONFLICT OF INTEREST

The authors declare no conflict of interest.

## Data Availability

All the data that support the findings of this study are available from the corresponding author upon reasonable request.
