# Supplementary material for: Cerebral organoids transplantation improves neurological motor function in rat brain injury
Source: CNS Neurosci Ther. 2020 Feb 22;26(7):682–97. doi: 10.1111/cns.13286 (PMC7298981; doi:10.1111/cns.13286)
Supplement: Supplementary file 1 [file CNS-26-682-s001.docx]

**Supplementary Material**

**Cerebral organoids transplantation improves neurological motor function in rat brain injury**

Zhi Wang^1, #^, Shu-Na Wang^1, #^, Tian-Ying Xu^1^, Chen Hong^1^, Ming-He Cheng^1^, Peng-Xi Zhu^1^, Jian-Sheng Lin^2^, Ding-Feng Su^1^, Chao-Yu Miao^1,^ *

^1^Department of Pharmacology, Second Military Medical University, Shanghai, China

^2^ Integrative Physiology of the Brain Arousal Systems, CRNL, INSERM U1028-CNRS UMR 5292, School of Medicine, Claude Bernard University, Lyon, France

***Corresponding Author:**

Chao-Yu Miao

MD, PhD, Professor and Chair

Department of Pharmacology

Second Military Medical University

325 Guo He Road, Shanghai 200433, China

Email: [cymiao@smmu.edu.cn](mailto:cymiao@smmu.edu.cn)

Tel: +86 21 81871271, Fax: +86 21 65493951

**Supplementary material includes seven figures and one table:**

**Supplementary Figure S1** COs transplantation improves neurogenesis and 55 d-CO promotes more neurogenesis than 85 d-CO in ipsilateral SGZ of rat TBI model

**Supplementary Figure S2** COs transplantation improves neurogenesis and 55 d-CO promotes more neurogenesis than 85 d-CO in ipsilateral SVZ of rat TBI model

**Supplementary Figure S3** COs transplantation has no impact on neural apoptosis in rat TBI model

**Supplementary Figure S4** COs transplantation has no impact on neuroinflammation in rat TBI model

**Supplementary Figure S5** Vascularization between transplanted COs and host brain of rat TBI model

**Supplementary Figure S6** Cells from transplanted COs migrate into cortex, thalamus and hippocampus along corpus callosum in rat TBI model

**Supplementary Figure S7** Cells from transplanted COs migrate into SVZ in rat TBI model

**Supplementary Table S1** Antibodies used in this study


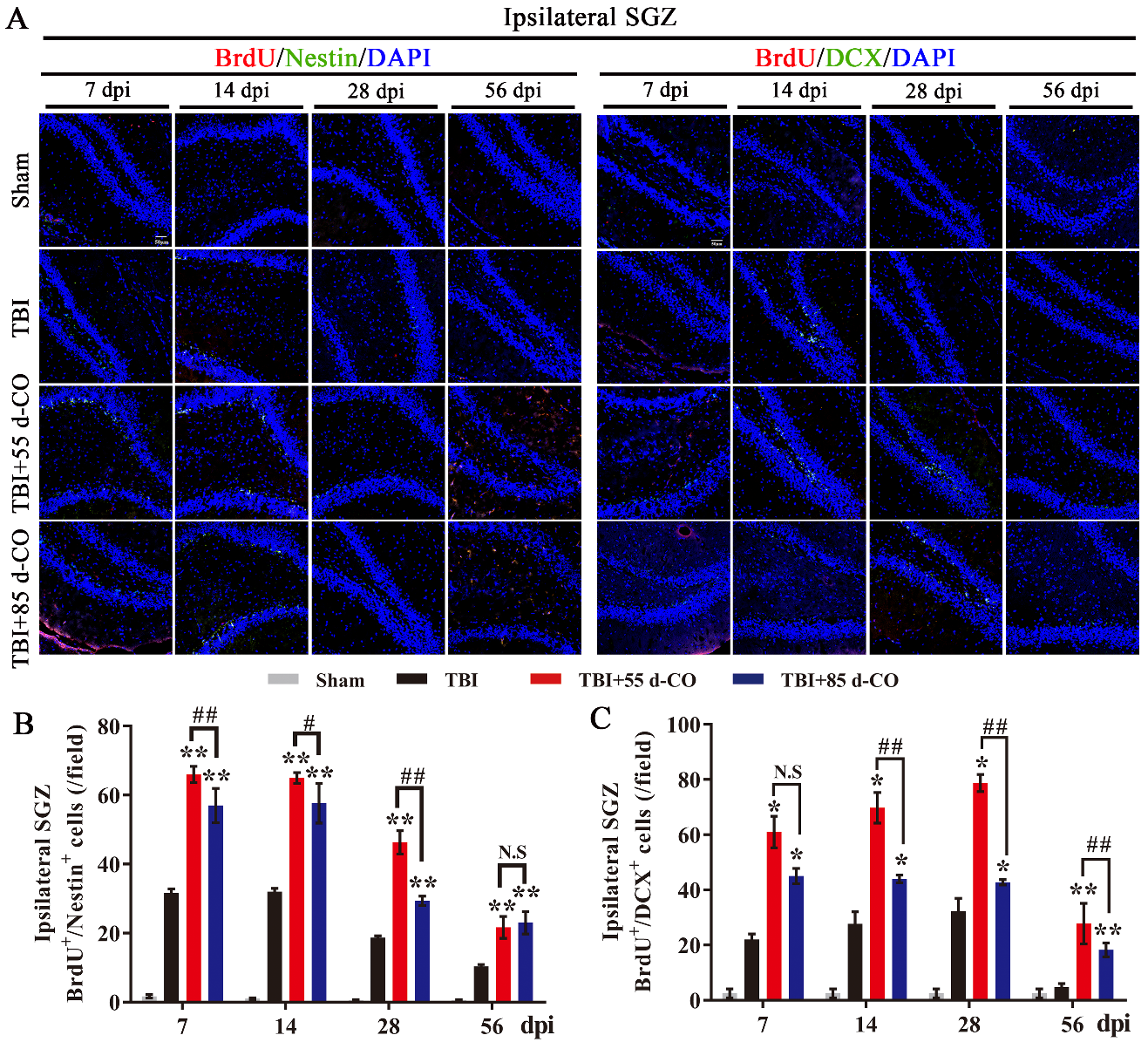


**Supplementary Figure S1 COs transplantation improves neurogenesis and 55 d-CO promotes more neurogenesis than 85 d-CO in ipsilateral SGZ of rat TBI model**

(**A**) Representative images of neurogenesis in rat ipsilateral SGZ of Sham, TBI, 55 d-CO transplantation and 85 d-CO transplantation groups by immunostaining of proliferated neural stem cells (BrdU^+^/Nestin^+^, red and green respectively) and migrated newborn neurons (BrdU^+^/DCX^+^, red and green respectively) at 7, 14, 28 and 56 dpi. DAPI labels nuclei (blue). Scale bars: 50 μm. (**B-C**) Quantitative analysis of neurogenesis by counting BrdU^+^/Nestin^+^ and BrdU^+^/DCX^+^ cells in rat ipsilateral SGZ at 7, 14, 28 and 56 dpi. Immuno-stained positive cells were counted with six random microscope fields of ipsilateral SGZ, and repeated with at least 3 independent animals per group. All data are shown as mean ± SEM and analyzed by ANOVA with Bonferroni posthoc tests. ^*^P < 0.05, ^**^P < 0.01 versus TBI group; ^#^P < 0.05, ^##^P < 0.01. N.S, not significant.


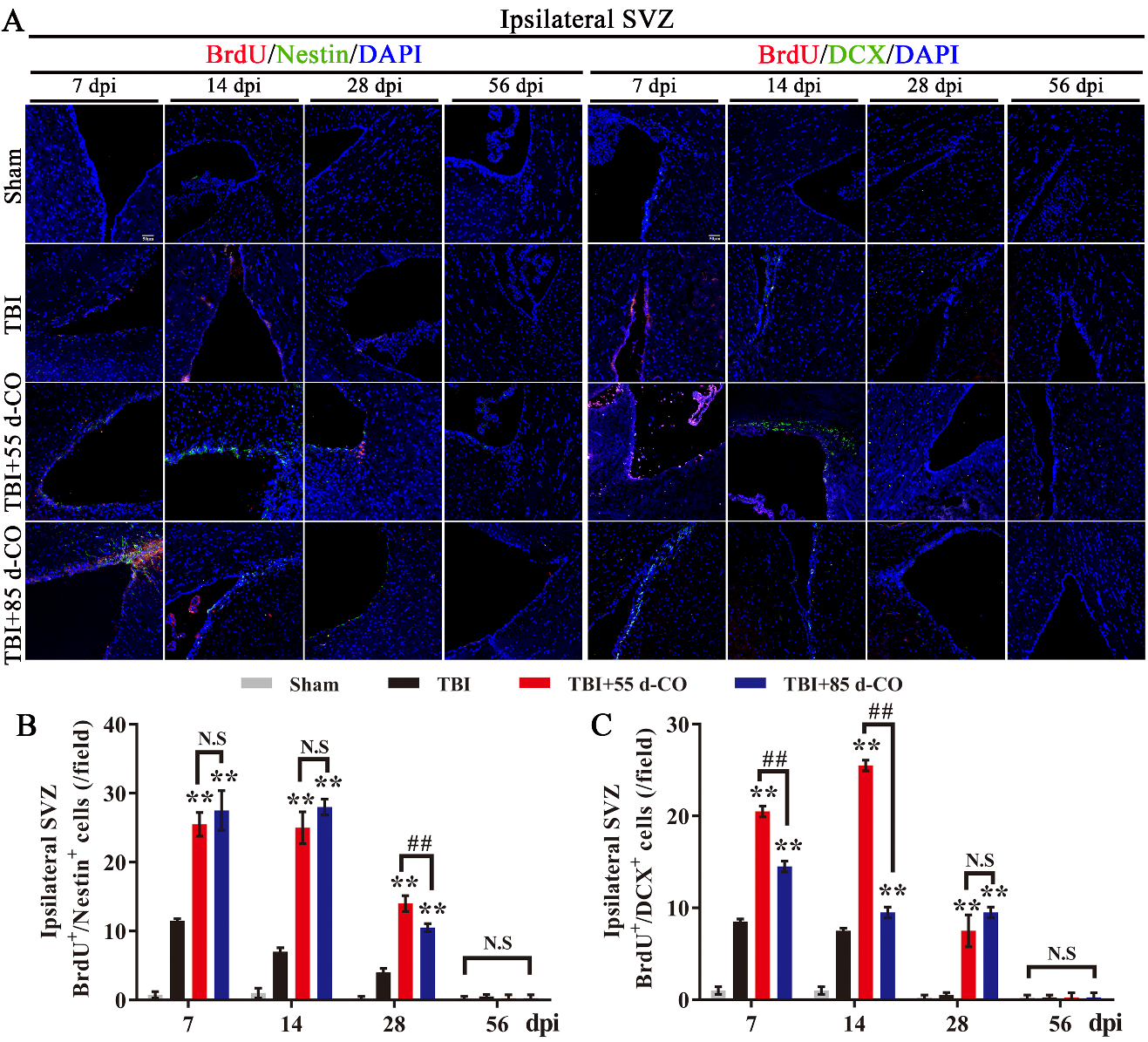


**Supplementary Figure S2 COs transplantation improves neurogenesis and 55 d-CO promotes more neurogenesis than 85 d-CO in ipsilateral SVZ of rat TBI model**

(**A**) Representative images of neurogenesis in rat ipsilateral SVZ of Sham, TBI, 55 d-CO transplantation and 85 d-CO transplantation groups by immunostaining of proliferated neural stem cells (BrdU^+^/Nestin^+^, red and green respectively) and migrated newborn neurons (BrdU^+^/DCX^+^, red and green respectively) at 7, 14, 28 and 56 dpi. DAPI labels nuclei (blue). Scale bars: 50 μm. (**B-C**) Quantitative analysis of neurogenesis by counting BrdU^+^/Nestin^+^ and BrdU^+^/DCX^+^ cells in rat ipsilateral SVZ at 7, 14, 28 and 56 dpi. Immuno-stained positive cells were counted with six random microscope fields of ipsilateral SVZ, and repeated with at least 3 independent animals per group. All data are shown as mean ± SEM and analyzed by ANOVA with Bonferroni posthoc tests. ^*^P < 0.05, ^**^P < 0.01 versus TBI group; ^#^P < 0.05, ^##^P < 0.01. N.S, not significant.


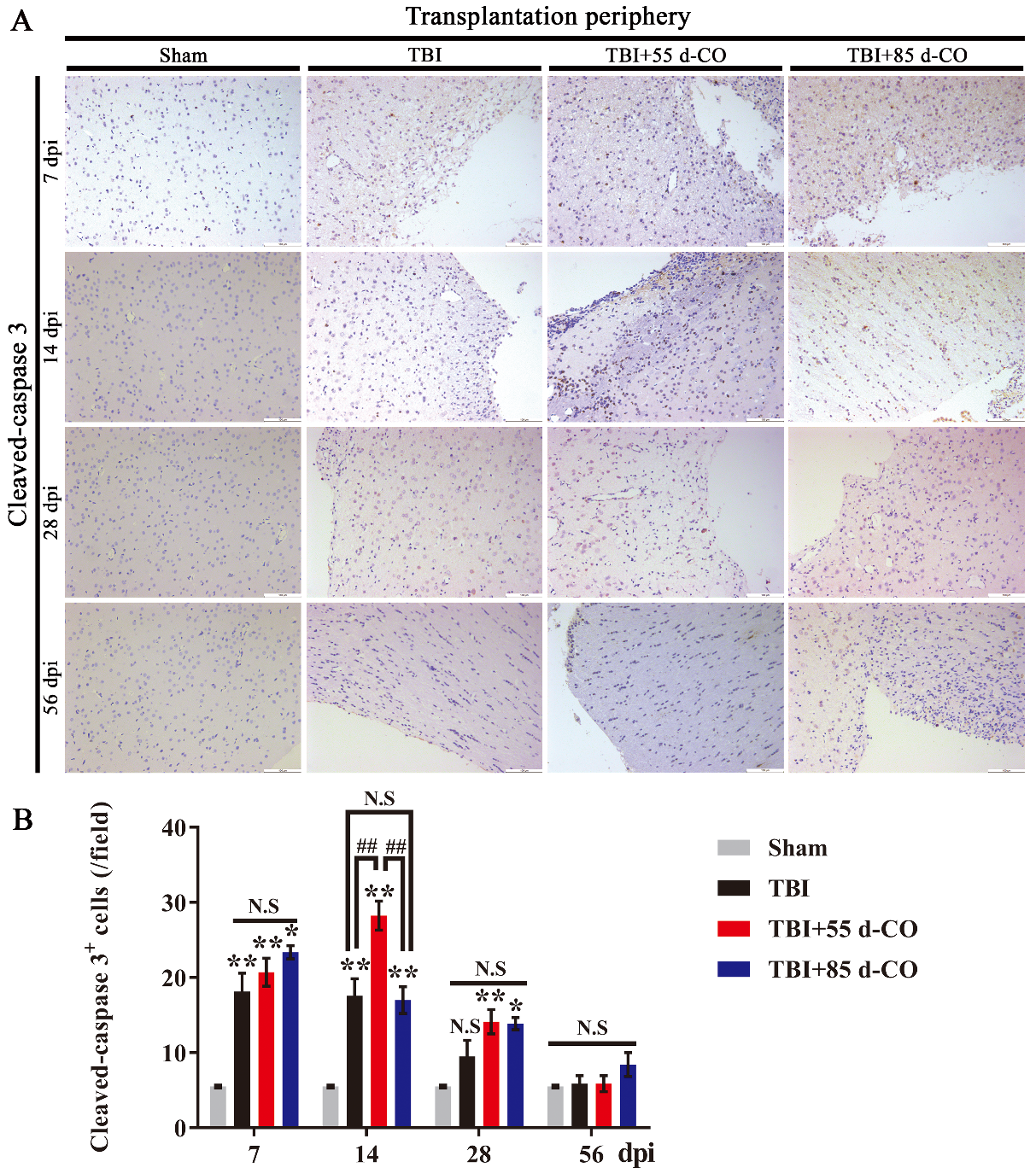


**Supplementary Figure S3 COs transplantation has no impact on neural apoptosis in rat TBI model**

(**A**) Representative images of apoptotic cells in transplantation periphery of ipsilateral cortex of Sham, TBI, 55 d-CO transplantation and 85 d-CO transplantation groups by immunostaining of cleaved-caspase 3 at 7, 14, 28 and 56 dpi. Scale bars: 100 μm. (**B**) Quantitative analysis of cleaved-caspase 3^+^ cells per field in the transplantation periphery of ipsilateral cortex. Cleaved-caspase 3^+^ cells were counted with six random microscope fields in transplantation periphery of ipsilateral cortex, and repeated with at least 3 independent animals per group. All data are shown as mean ± SEM and analyzed by ANOVA with Bonferroni posthoc tests. *P < 0.05, **P < 0.01 versus Sham group; ##P < 0.01. N.S, not significant.


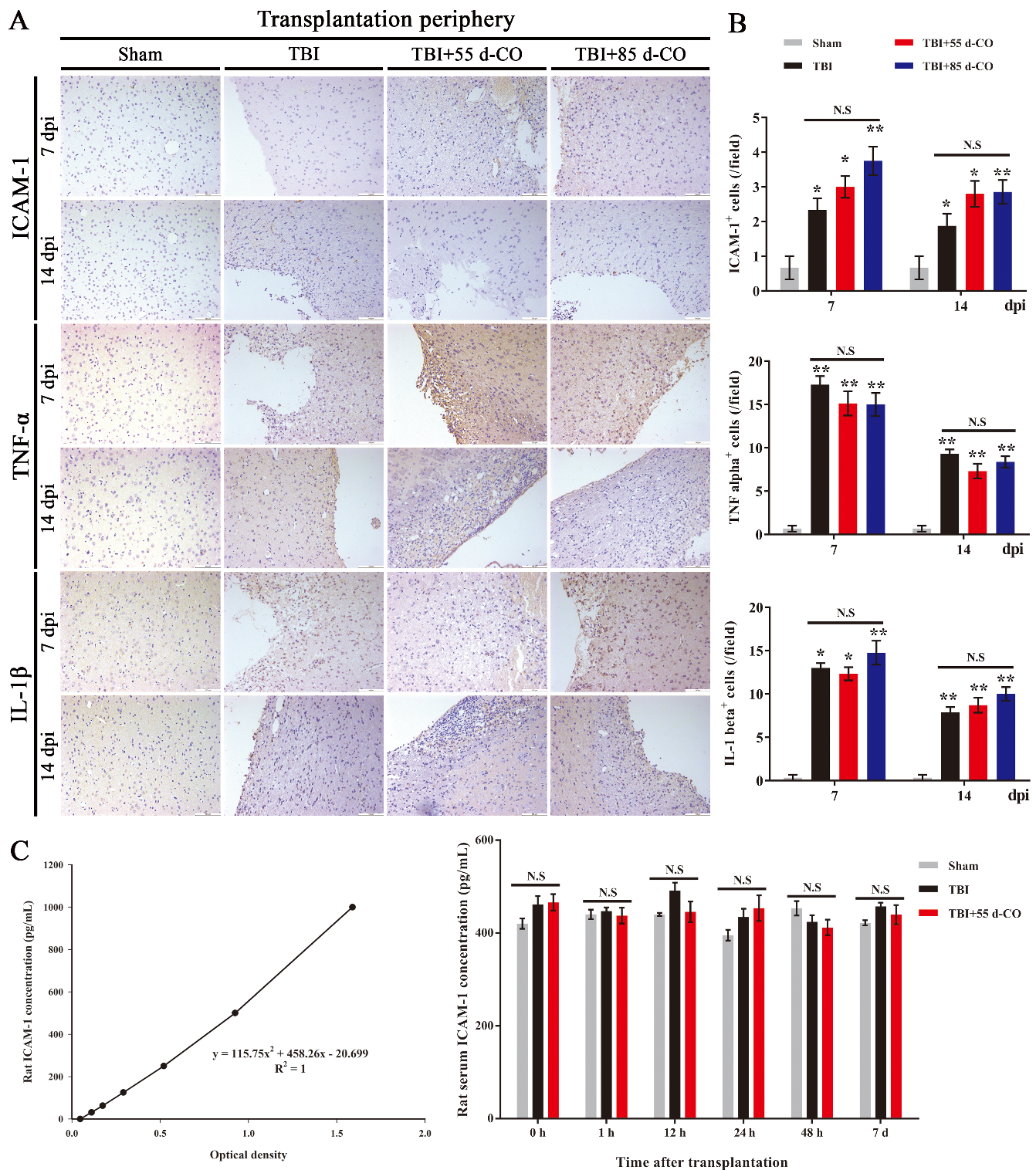


**Supplementary Figure S4 COs transplantation has no impact on neuroinflammation in rat TBI model**

(**A**) Representative images of neuroinflammation in transplantation periphery of ipsilateral cortex of Sham, TBI, 55 d-CO transplantation and 85 d-CO transplantation groups by immunostaining of intercellular cell adhesion molecule-1 (ICAM-1), tumor necrosis factor α (TNF-α) and interleukin-1 β (IL-1β) at 7 and 14 dpi. Scale bars: 100 μm. (**B**) Quantitative analysis of ICAM-1^+^, TNF-α^+^ and IL-1β^+^ cells in transplantation periphery of ipsilateral cortex at 7 and 14 dpi. Immuno-stained positive cells were counted with six random microscope fields in transplantation periphery of ipsilateral cortex, and repeated with at least 3 independent animals per group. (**C**) Rat ICAM-1 serum level in rat TBI model. The left curve is the standard curve of rat ICAM-1 ELISA kit. The right histogram is the rat serum ICAM-1 concentration in Sham, TBI and TBI transplanted with 55 d-CO groups. There was no difference among Sham, TBI and 55 d-CO transplantation groups. All data are shown as mean ± SEM and analyzed by ANOVA with Bonferroni posthoc tests. ^*^P < 0.05, ^**^P < 0.01 versus Sham group. N.S, not significant.


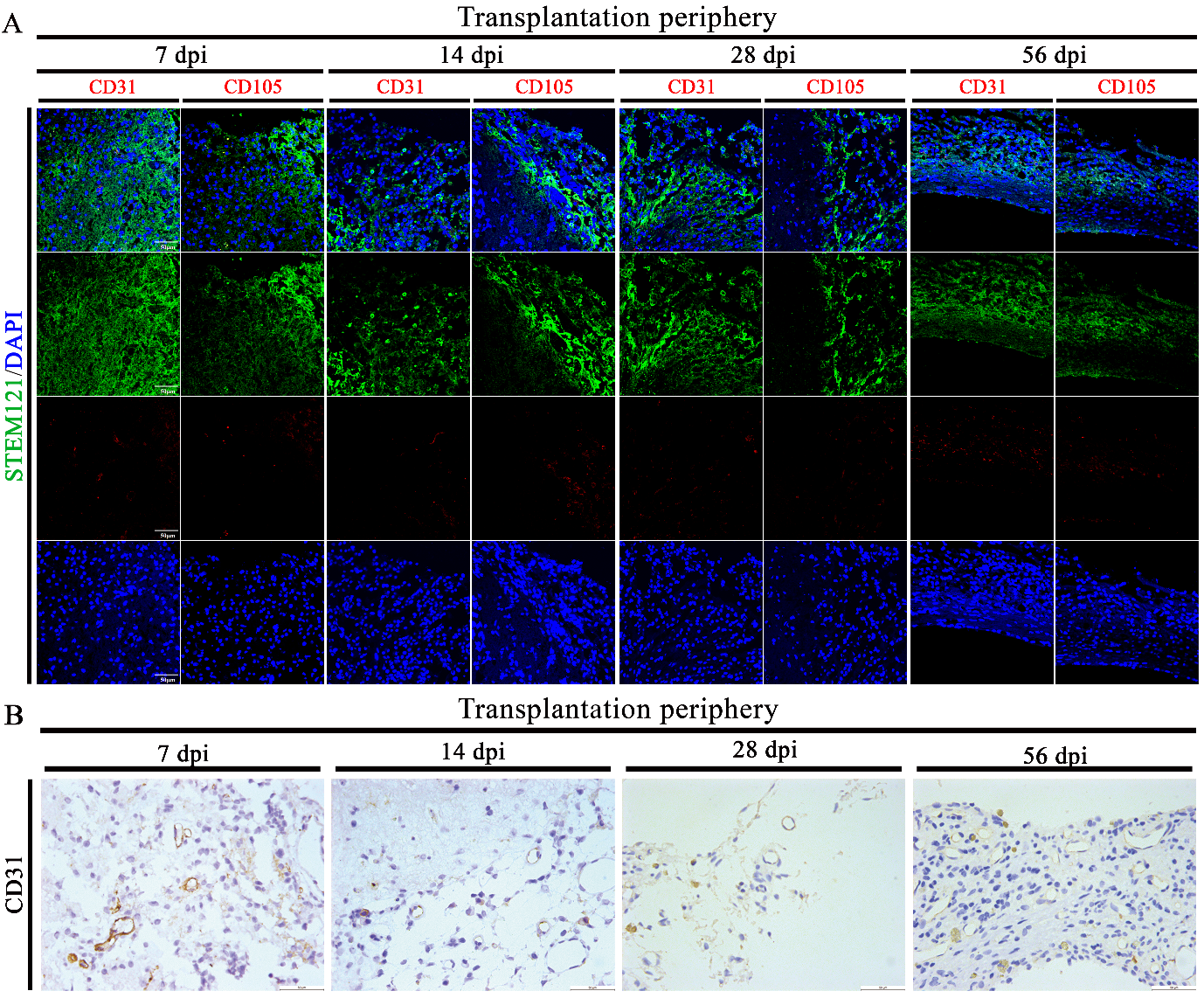


**Supplementary Figure S5 Vascularization between transplanted COs and host brain of rat TBI model**

(**A**) Representative images of vascularization between transplanted COs and host brain by immunostaining of human cytoplasmic marker STEM121 (green) with endothelial cells marker CD31 (red) or CD105 (also endoglin, red) at 7, 14, 28 and 56 dpi. DAPI labels nuclei (blue). Scale bars: 50 μm. (**B**) Representative images of vascular formation between transplanted COs and host brain by immunostaining of CD31^+^ endothelial cells. Scale bars: 50 μm.


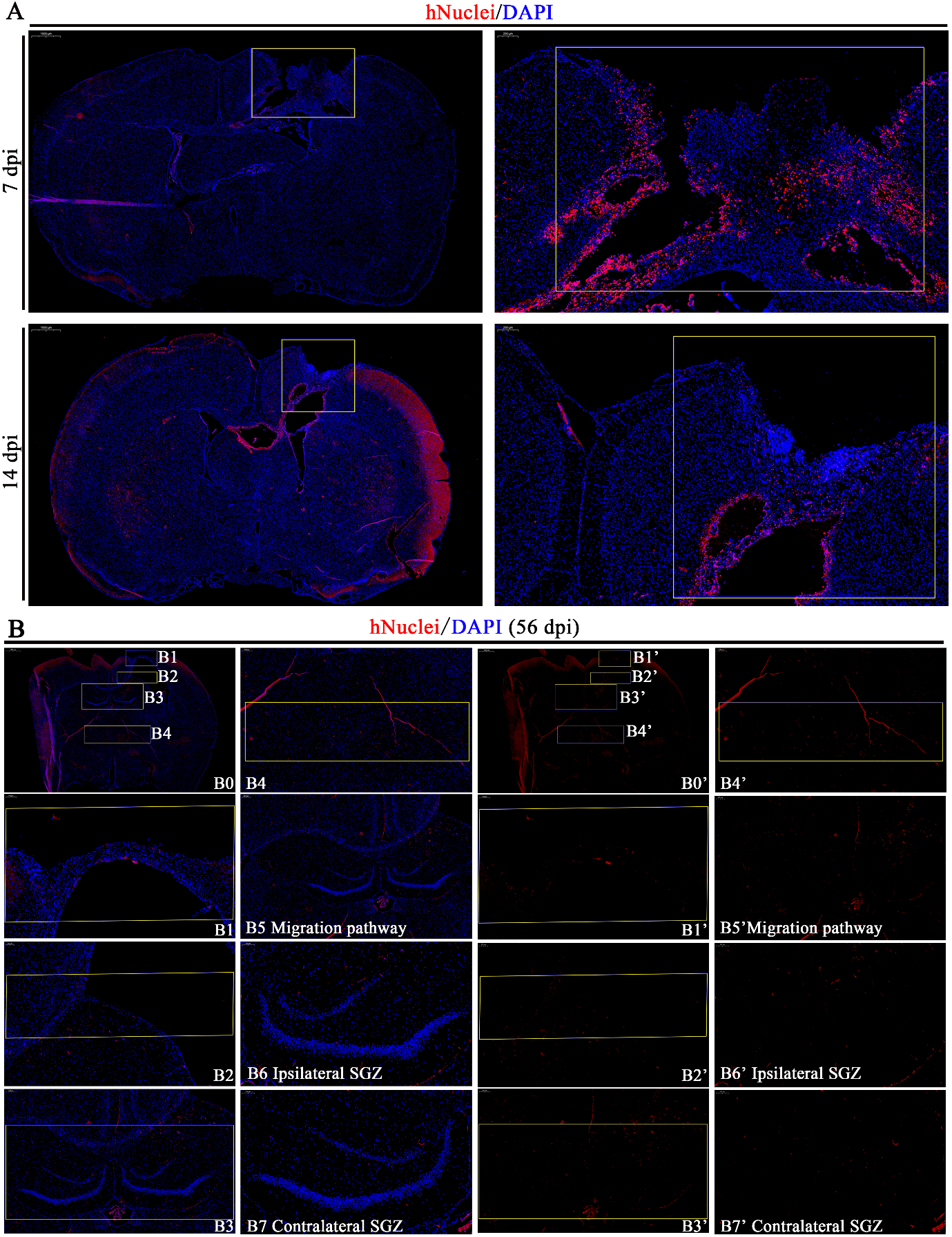


**Supplementary Figure S6 Cells from transplanted COs migrate into cortex, thalamus and hippocampus along corpus callosum in rat TBI model**

(**A**) Representative images of whole brain scan with COs transplantation by immunostaining of human nuclear marker (hNuclei, red) at 7 and 14 dpi in 55 d-CO transplantation group. Tissues inside the rectangle frame indicate the transplanted COs in host brain, wherein the right image is the high-magnification view of boxed area in the left image. (**B**) Representative images of migration of cells from transplanted COs into host brain at 56 dpi in 55 d-CO transplantation group. (**B0 and B0’**) Overall view of migration of cells from transplanted COs into rat brain. (**B2 and B2’, B5 and B5’**) Images showed corpus callosum as migration pathway of human cells into host brain. Cells from transplanted COs showed migration and integration into cortical region (**B1 and B1’**), and migration into ipsilateral and contralateral hippocampus (**B3 and B3’**), thalamic nucleus (**B4 and B4’**), ipsilateral and contralateral SGZ (**B6 and B6’, B7 and B7’**) in the host brain. DAPI labels nuclei (blue).


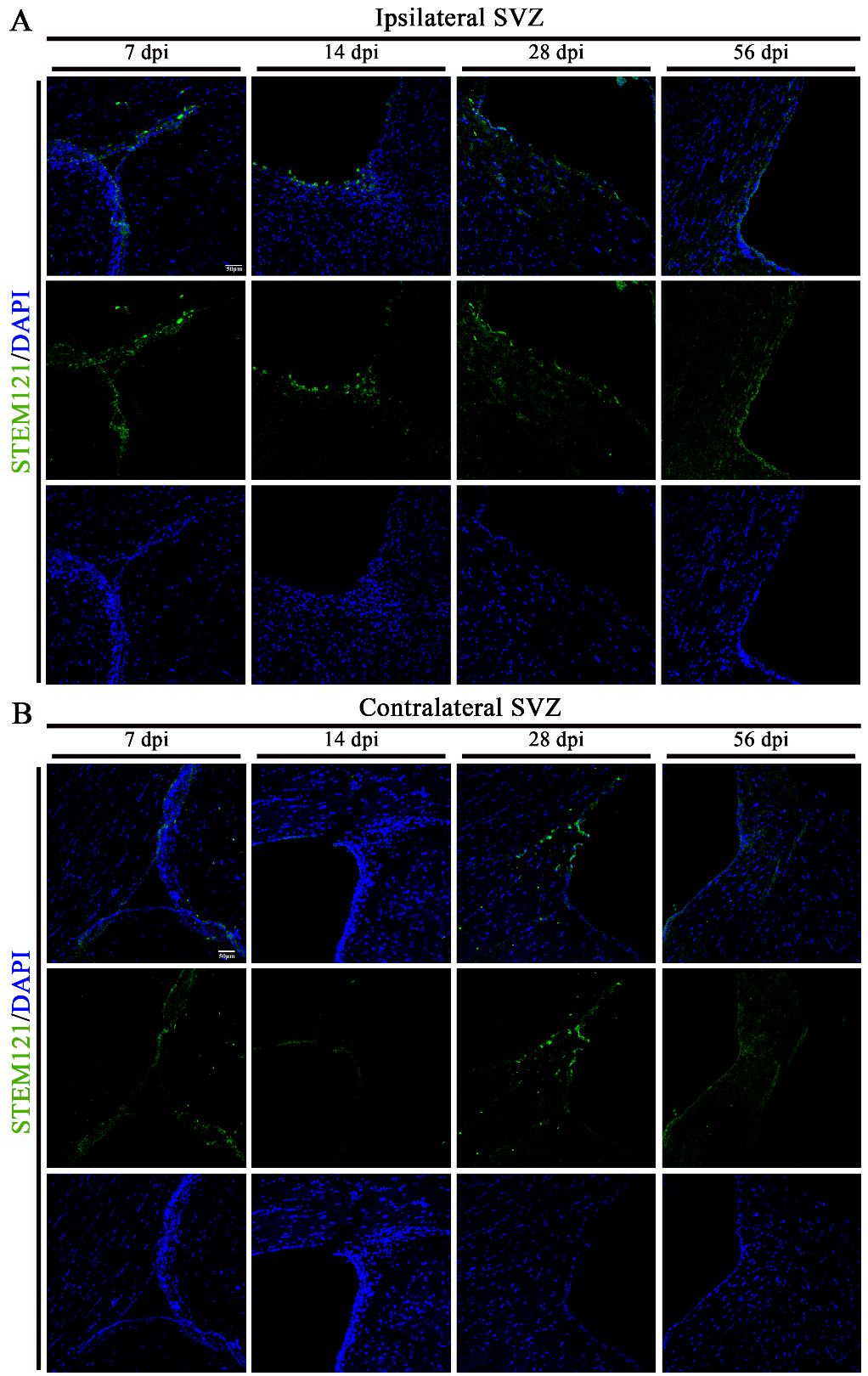


**Supplementary Figure S7 Cells from transplanted COs migrate into SVZ in rat TBI model**

(**A and B**) Representative images of cells from transplanted COs in ipsilateral and contralateral SVZ by immunostaining of human cytoplasmic marker (STEM121, green) at 7, 14, 28 and 56 dpi. Cells from transplanted COs migrate into rat SVZ of host brain. DAPI labels nuclei (blue). Scale bars: 50 μm.

**Supplementary Table S1 Antibodies used in this study**

| **Antibodies** | **Expression** | **Host** | **Reactivity** | **Source/Cat.NO.** | **Dilution** |
| --- | --- | --- | --- | --- | --- |
| SOX2 | Neural progenitor cell | Rb | H, R | Abcam (ab97959) | 1:250 (ICC/IF) |
| Nestin | Neural stem cell | M | H, R | Abcam (ab6142) | 1:200 (ICC/IF) |
| Tuj1 | Neuron | Rb | H, R | CST (5568s) | 1:200 (ICC/IF) |
| NeuN | Mature neuron | Rb | H, R | Abcam (ab177487) | 1:300 (ICC/IF) |
| DCX (Doublecortin) | Newborn neuron | Rb | H, R | CST (4604S) | 1:400 (ICC/IF) |
| GFAP | Astrocyte | Rb | H, R | Abcam (ab7260) | 1:300 (ICC/IF) |
| BrdU | Proliferation cell | S | / | Abcam (ab1893) | 1:200 (ICC/IF) |
| Foxg1 | Forebrain | Rb | H, R | Abcam (ab18259) | 1:250 (ICC/IF) |
| TTR (Prealbumin) | Choroid plexus | S | H, R | Abcam (ab9015) | 1:250 (ICC/IF) |
| Chat | choline acetyltransferase enzyme | Rb | H, R | Abcam (ab181023) | 1:250 (ICC/IF) |
| Olig2 | oligodendrocyte and motor neuron progenitor cell | Rb | H, R | Abcam (ab136253) | 1:250 (ICC/IF) |
| TBR1 | pre-plate/deep-layer neuron | M | H, R | Santa Cruze (SC-376258) | 1:250 (ICC/IF) |
| SATB2 | surface-layer neuron | M | H, R | Santa Cruze (SC-81376) | 1:250 (ICC/IF) |
| vGlut1 | glutamatergic neuron | M | H, R | Santa Cruze (sc-377425) | 1:250 (ICC/IF) |
| STEM121 | Human cytoplasmic marker | M | H | Takara (Y40410) | 1:200 (ICC/IF) |
| hNuclei | Human nuclear marker | Rb | H | Abcam (ab108595) | 1:250 (ICC/IF) |
| CD31 | Endothelial cell | Rb | R | Abcam (ab222783) | 1:100 (ICC/IF) |
| CD31 | Endothelial cell | Rb | H, R | Abcam (ab182981) | 1:100 (IHC) |
| CD105 | Endothelial cell | Rb | H, R | Abcam (ab107595) | 1:100 (ICC/IF) |
| Cleaved-caspase 3 | Apoptotic cell | Rb | H, R | Abcam (ab13847) | 1:300 (IHC) |
| ICAM-1 | Intercellular cell adhesion molecule-1 | M | R | Abcam (ab171123) | 1:100 (IHC) |
| TNFα | Tumor necrosis factor α | Rb | R | Abcam (ab6671) | 1:200 (IHC) |
| IL-1β | Interleukin-1 β | Rb | R | Abcam (ab9787) | 1:200 (IHC) |
| Synaptophysin | Presynaptic marker | Rb | H, R | Abcam (ab14692) | 1:500 (WB) |
| PSD 95 | Postsynaptic marker | Rb | R | CST (3919S) | 1:1000 (WB) |
| BDNF | Brain derived neurotrophic factor | M | H, R | Abcam (ab205067) | 1:500 (WB) |
| EGF | Epidermal growth factor | Rb | R | Abcam (ab184265) | 1:500 (WB) |
| NGF | Nerve growth factor | Rb | H, R | Abcam (ab52918) | 1:500 (WB) |

M: mouse; R: rat; Rb: rabbit; S: sheep; H: human. ICC: immunocytochemistry; IF: immunofluorescence; IHC: immunohistochemistry; WB: Western blot.
